# Candidate inflammatory biomarkers display unique relationships with alpha-synuclein and correlate with measures of disease severity in subjects with Parkinson’s disease

**DOI:** 10.1186/s12974-017-0935-1

**Published:** 2017-08-18

**Authors:** Lori N. Eidson, George T. Kannarkat, Christopher J. Barnum, Jianjun Chang, Jaegwon Chung, Chelsea Caspell-Garcia, Peggy Taylor, Brit Mollenhauer, Michael G. Schlossmacher, Larry Ereshefsky, Mark Yen, Catherine Kopil, Mark Frasier, Kenneth Marek, Vicki S. Hertzberg, Malú G. Tansey

**Affiliations:** 1 Department of Physiology, Emory University, 615 Michael Street, 605L Whitehead Biomedical Res. Bldg., Atlanta, GA 30322 USA; 2 Department of Biostatistics, University of Iowa, 145 N. Riverside Drive, 100 CPHB, Iowa City, Iowa, 52242 USA; 3 BioLegend, Inc., 180 Rustcraft Rd # 140, Dedham, Massachusetts 02026 USA; 4 Paracelsus-Elena-Klinik, 34128 Kassel, Kassel, Germany; 5 Georg-August University Medical Center Goettingen, 37075 Goettingen, Germany; 6 Program in Neuroscience and Division of Neurology, The Ottawa Hospital, University of Ottawa Brain & Mind Institute, 451 Smyth Road, Room 1412, Ottawa, K1H 8M5 Canada; 7 Follow the Molecule, 143 Voyage Mall, Marina del Rey, CA 90292 USA; 8 PAREXEL International, Early Phase Unit, 1560 E. Chevy Chase Drive, Suite 140, Glendale, CA 91206 USA; 9 Research Programs, The Michael J. Fox Foundation for Parkinson’s Research, 69 7th Avenue, 498, New York, NY 10018 USA; 10 Yale-New Haven Hospital, 20 York Street, New Haven, CT 06510 USA; 11 Nell Hodgson Woodruff School of Nursing, Emory University, 1520 Clifton Rd, Atlanta, GA 30322 USA

**Keywords:** Parkinson’s disease, Inflammation, Protein biomarkers, Daily rhythm, CSF, Serum

## Abstract

**Background:**

Efforts to identify fluid biomarkers of Parkinson’s disease (PD) have intensified in the last decade. As the role of inflammation in PD pathophysiology becomes increasingly recognized, investigators aim to define inflammatory signatures to help elucidate underlying mechanisms of disease pathogenesis and aid in identification of patients with inflammatory endophenotypes that could benefit from immunomodulatory interventions. However, discordant results in the literature and a lack of information regarding the stability of inflammatory factors over a 24-h period have hampered progress.

**Methods:**

Here, we measured inflammatory proteins in serum and CSF of a small cohort of PD (*n* = 12) and age-matched healthy control (HC) subjects (*n* = 6) at 11 time points across 24 h to (1) identify potential diurnal variation, (2) reveal differences in PD vs HC, and (3) to correlate with CSF levels of amyloid β (Aβ) and α-synuclein in an effort to generate data-driven hypotheses regarding candidate biomarkers of PD.

**Results:**

Despite significant variability in other factors, a repeated measures two-way analysis of variance by time and disease state for each analyte revealed that serum IFNγ, TNF, and neutrophil gelatinase-associated lipocalin (NGAL) were stable across 24 h and different between HC and PD. Regression analysis revealed that C-reactive protein (CRP) was the only factor with a strong linear relationship between CSF and serum. PD and HC subjects showed significantly different relationships between CSF Aβ proteins and α-synuclein and specific inflammatory factors, and CSF IFNγ and serum IL-8 positively correlated with clinical measures of PD. Finally, linear discriminant analysis revealed that serum TNF and CSF α-synuclein discriminated between PD and HC with a minimum of 82% sensitivity and 83% specificity.

**Conclusions:**

Our findings identify a panel of inflammatory factors in serum and CSF that can be reliably measured, distinguish between PD and HC, and monitor inflammation as disease progresses or in response to interventional therapies. This panel may aid in generating hypotheses and feasible experimental designs towards identifying biomarkers of neurodegenerative disease by focusing on analytes that remain stable regardless of time of sample collection.

**Electronic supplementary material:**

The online version of this article (doi:10.1186/s12974-017-0935-1) contains supplementary material, which is available to authorized users.

## Background

A growing body of literature supports a role for peripheral and central immune cells and inflammation in the pathogenesis and progression of neurodegenerative diseases including Parkinson’s disease (PD) [1–5]. Yet the major focus in identification of biomarkers for Parkinson’s disease (PD) to date has focused solely on neuronal proteins (e.g., α-synuclein, tau, and β-amyloid) because these proteins are known to play a role in the fundamental pathophysiology of neurodegenerative diseases; and in PD patients, the levels of α-synuclein have been found to be decreased in the CSF compared to that of HC individuals [6], a phenotype which does not change significantly within the first months after diagnosis [7] and suggests these proteins are not being efficiently removed from the brain parenchyma. However, inflammatory markers of disease have gained interest as potential, earlier indicators of neurodegenerative disease processes and may have a predictive value. Age is the most common risk factor for development of PD; like other immune cells, microglia display age-dependent changes in activation and regulation [8]. Post mortem analysis of CSF and brain tissue consistently indicates the presence of activated microglia, increased levels of cytokines likely to be microglial-derived, increased NFkB activation, and oxidative damage at autopsy [9–12]. Brain imaging of live subjects confirms increased inflammation in the pons, basal ganglia, striatum, frontal cortex, and temporal cortex in PD [13–15], and pre-clinical animal models [16–24], and clinical studies [9, 10] demonstrate that inflammation-derived oxidative stress and cytokine-dependent toxicity contribute to nigrostriatal pathway degeneration [3, 25, 26]. These inflammatory factors are considered to derive from chronically activated microglia and invading immune cells responding to aggregation of toxic α-synuclein oligomers, early neuronal dysfunction [25], and dying neurons in later disease stages [2], and play a pivotal role in the initiation and propagation of illness.

Although there is no consensus on the role of inflammation as a primary, causative, or early factor in PD or a secondary byproduct of disease, and the role of inflammation in PD is still a hypothesis, there is a great interest in establishing which inflammatory markers can help stage disease and identify disease endophenotypes, to inform immunomodulatory interventions, as has been effectively demonstrated in multiple sclerosis [27], for example. To this end, several studies have sampled biofluids for correlation with disease state. However, these data are conflicting and difficult to interpret. A recent meta-analysis [28] revealed that not all studies indicate elevation of key inflammatory factors but most report significant differences between PD and healthy controls, suggesting that PD is accompanied by a dysregulated inflammatory response. Specifically, several studies reported increased inflammation in CSF [29–32] and serum [33] of PD subjects, but there is disagreement regarding direction of change for several markers. For instance, serum IFNγ has been reported to be increased [33], decreased [34], and not different [28] in PD subjects at various stages compared with HCs. Similarly, serum TNF has been reported to be increased in PD subjects compared with age-matched HC subjects [33–37], but decreased serum TNF levels [38] have also been reported. These disparities may be due to differences in disease severity, other comorbidities, different operating protocols, individual variability, differences in sample processing, analytical methodologies, and almost certainly differences in sampling times coupled with diurnal fluctuations of inflammatory proteins.

Herein, we describe CSF and serum inflammation at 11 time points across a 24-h period (spanning in effect 26 h) in PD patients and age-matched HC subjects in an effort to identify a subset of readily detectable and stable inflammatory factors. The first part of this study evaluated the stability of key CSF biomarkers, alpha-synuclein, DJ-1, and Abeta1-42 in young healthy volunteers in a two period study using the same sampling schedule as outlined here. A minimum of two weeks separated the repeat sampling for blood and CSF [43]. Given that endogenous cortisol levels peak in the morning [39] and can affect cytokine levels [40, 41], we hypothesized that a subset of inflammatory factors in central and/or peripheral compartments display normal diurnal variations in HC subjects and these may be disrupted in patients with PD. We further hypothesize that a subset of inflammatory markers does not display diurnal variability and may be different between HC and PD subjects and represent potential disease-specific inflammatory markers. Our goals were to identify the variability in potential candidate biomarkers of inflammation to power larger studies and generate hypotheses, to examine relationships between CSF and serum inflammation for each inflammatory factor, to examine associations between peripheral and central inflammatory factors and CSF levels of α-synuclein, β-amyloid 1–40 (Aβ_40_), and 1–42 (Aβ_42_), and to define an ideal set of inflammatory factors in serum and CSF to be used in conjunction with levels of CSF α-synuclein and Aβ proteins to distinguish PD vs HC subjects with sensitivity and specificity.

## Methods

### Subject inclusion/exclusion criteria

A total of 18 PD and 8 HC subjects were screened; of these, 12 subjects with PD (as diagnosed by a movement disorder-trained physician based on the widely employed criteria [42]) and 6 age-matched HC subjects were included. All study participants completed the study except one PD subject who dropped out of CSF collection due to minor discomfort. There were no significant differences in age, weight, or body mass index (BMI) between the PD and HC groups. PD subjects must have had at least two of the following symptoms: resting tremor, bradykinesia, or rigidity (either resting tremor or bradykinesia), a diagnosis of PD for ≤ 10 years, and a Hoehn and Yahr (H&Y) stage of I–III. HC subjects with current clinically significant neurological disorder and/or a first-degree relative with idiopathic PD were excluded. Twenty-eight days prior to sample collection and 1 day prior to sample collection, subjects underwent physical and neurological examination, Unified Parkinson’s Disease Rating Scale (UPDRS) assessment, H&Y assessment, spinal x-ray (unless taken within the last 12 months), vital signs, medical and medication history, 12–lead electrocardiogram (ECG), safety laboratory assessments, coagulation screening, urine drug screening, urine ethanol screening, Hepatitis B and C screening, HIV testing, and serum pregnancy testing (females). Study participants were not taking prescription or non-prescription drugs within 7 days or 5 half lives (whichever is longer) of sample collection, with the exception of PD subjects taking a stable dose (for 4 or more weeks) of PD medication (amantadine, dopamine agonists, L-DOPA, and/or MAO-B inhibitors) prior to sample collection.

### Study design

Subjects were admitted to the clinic the day prior to sample collection for baseline assessments, as described above. Lumbar and venous catheters were inserted, and CSF and blood were collected concurrently over 26 h (i.e., at ~ 5:30 AM, time 0; within 30 min of catheterization, 1, 2, 4, 6, 10, 12, 16, 20, 24, and 26 h post catheterization). Vital signs were taken regularly, and subjects remained in the clinic for at least 24 h following sample collections for monitoring, and a final neurological examination before discharge. Study procedures were safe and well tolerated with no serious adverse events related to the procedure [43].

### Sample collection and handling

The samples were accessed through the 24 h Biofluids bank, a subset of a large Michael J. Fox Foundation biospecimen bank available to the community (https://www.michaeljfox.org/page.html?id=193&navid=data-biospecimens). CSF samples were collected via intrathecal catheter connected to a peristaltic roller pump. Sample collection (6 ml) started approximately 12 min before each identified sampling time and was concluded approximately 6 min after. The catheter was cleared of any residual CSF before sample collections to ensure fresh sampling. CSF samples were centrifuged at × 1600*g* for 15 min at 4 °C, supernatants were transferred into six 0.5 mL-aliquots and three 1.0 mL-aliquots in polypropylene tubes, and stored at − 80 °C or on dry ice within 1 h of collection. CSF samples contaminated with blood were discarded. The quality of the CSF samples was visually colorless, and red blood cell (RBC) analysis from the 2-ml discard sample was conducted at each time point. The analysis was conducted within 1 h of sample collection. Whole blood (10 ml) was collected in red top vacutainers, centrifuged at × 1350*g* for 15 min at 4 °C, serum was transferred into six 0.5 mL-aliquots and three 1.0 mL-aliquots, stored in polypropylene cryotubes, and stored at − 80 °C until shipment on dry ice. All subjects had CSF assessment for blood contamination (hemoglobin ELISA) as part of the safety assessments, typically twice during each catheterization period. In general, if not noted in the clinical study report, this would mean a clinically insignificant finding for RBCs in ~  98% of the samples.

### ELISA measurements for α-synuclein, Aβ proteins, and hemoglobin

α-Synuclein concentration was determined using an ELISA with a luminescent readout (Covance SIG-38974, now BioLegend 8441010 [44]. All samples were run in duplicate at 1:10, 1:20, and 1:50. The reference standards ranged from 6.1–1500 pg/ml. Concentrations of α-synuclein were determined by comparing to the reference standard curve using a 4-parameter regression. Recombinant α-synuclein from rPeptide was used as the standard. Aβ_1–42_ was measured using the Betamark Chemilumiescent Assay (Covance, SIG-38952), Aβ_1–40_ was detected using the Covance Sandwich ELISA (Covance, SIG-38950), total protein was determined using a BCA protein assay (ThermoFisher/Pierce Bio), and hemoglobin was measured by sandwich ELISA (Bethyl Laboratories) according to manufacturer’s instructions.

### Multiplexed immunoassay measurements of inflammatory proteins

Inflammatory proteins were measured using the Meso Scale Discovery (MSD) electrochemiluminescent (ECL) immunoassay V-PLEX human pro-inflammatory panel (i.e., IL-1β; detection range: 0.04–1000 pg/ml, IL-2; 0.09–1000 pg/ml, IL-6; 0.06–1000 pg/ml, IL-8; 0.04–1000 pg/ml, IL-4; 0.02–100 pg/ml, IL-10; 0.03–1000 pg/ml, IL-12p70; 0.1–1000 pg/ml, IL-13; 0.2–1000 pg/ml, IFNγ; 0.2–1000 pg/ml, and TNF; 0.04–100 pg/ml) and a V-PLEX human NGAL (1–10,000 pg/ml) and CRP (100–100,000 pg/ml) panel on the MSD SECTOR Imager 2400-A (Meso Scale Diagnostics, LLC, Rockville, MD) according to manufacturer’s instructions. CSF and serum samples were run in duplicate, and serum samples were diluted by a factor of 1. Data were analyzed by laboratory personnel blinded to condition using MSD integrated data analysis software that converts ECL signal to pg/mL values based on standard curves of calibrator proteins. All % coefficient of variation (CV) values for samples analyzed were below 5%.

### Statistical analysis and data presentation

As an ad hoc means of determining stability over time, we performed linear regression of each analyte over time for each individual. Inflammatory factors with no significant association with time were classified as “stable,” and factors with a significant association with time were classified as “positive” or “negative” according to the sign of the slope estimate (Additional file 1). A Mann-Whitney *U* rank-sum nonparametric test was used to compare levels of each analyte between HC and PD subjects at time 0 (Fig. 2 and Additional file 2). We used orthogonal polynomials to examine diurnal patterns, in particular the quadratic effect (Table 1). We applied repeated measures ANOVA to assess the effects of time and to examine differences between HD and PD participants (Fig. 2 and Additional file 3). We also investigated the extent to which levels of an inflammatory marker in serum correlated with its levels in the CSF (Additional file 4). We did not adjust for multiple comparisons given the low group sizes and the nature of this study being exploratory in order to be able to power a larger study. We used repeated measures ANOVA to examine the relationship between serum and CSF inflammatory factor levels across time for each subject, as well as those between serum and CSF inflammatory markers with CSF α-synuclein and Aβ proteins across time (Additional file 5). We used ANCOVA to determine these relationships at baseline (Additional file 6 **,** Fig. 3 **,** and Additional file 7) as well as relationships with UPDRS and its components (Fig. 4 **,** Additional file 8). Data are reported as the mean protein concentration (pg/mL) ± SEM. Finally, we used linear discriminant analysis (LDA) to determine the ability of biomarker groups to discriminate between HC and PD participants (Fig. 4 and Additional file 9). *p* ≤ 0.05 was considered significant.

**Table 1:** CSF TNF, CRP, IL-8, IL-6, α-synuclein, and Aβ levels were disrupted in PD versus HC

Quadratic effect	HC		PD	
Serum analyte	*F* statistics	*p* value	*F* statistics	*p* value
TNF	*F*_(1,5)_ = 0.06	0.82	*F*_(1,11)_ = 0.02	0.89
IFNγ	*F*_(1,5)_ = 0.48	0.52	*F*_(1,11)_ = 1.16	0.31
NGAL	*F*_(1,5)_ = 2.24	0.19	*F*_(1,11)_ = 1.37	0.27
CRP	*F*_(1,5)_ = 0.09	0.78	*F*_(1,11)_ = 3.62	0.08
IL-6	*F*_(1,5)_ = 5.37	0.07	*F*_(1,11)_ = 0.10	0.76
IL-8	*F*_(1,5)_ = 0.11	0.76	*F*_(1,11)_ = 2.88	0.12
CSF Analyte	*F* statistics	*p* value	*F* statistics	*p* value
TNF	*F*_(1,5)_ = 5.39	0.07	*F*_(1,10)_ = 7.47	0.02
IFNγ	*F*_(1,5)_ = 0.00	0.99	*F*_(1,10)_ = 1.24	0.29
NGAL	*F*_(1,5)_ = 0.16	0.71	*F*_(1,10)_ = 0.90	0.37
CRP	*F*_(1,5)_ = 1.67	0.25	*F*_(1,10)_ = 6.51	0.03
IL-6	*F*_(1,5)_ = 15.00	0.01	*F*_(1,10)_ = 2.97	0.12
IL-8	*F*_(1,5)_ = 1.28	0.31	*F*_(1,10)_ = 8.45	0.02
α-synuclein	*F*_(1,5)_ = 3.89	0.11	*F*_(1,11)_ = 6.02	0.03
Aβ_40_	*F*_(1,5)_ = 26.36	0.004	*F*_(1,11)_ = 8.19	0.02
Aβ_42_	*F*_(1,5)_ = 5.60	0.06	*F*_(1,11)_ = 11.60	0.006

An analysis of linear and quadratic trends indicated that levels of CSF inflammation rise and fall across the day in PD subjects more than HC. TNF, CRP, IL-8, α-synuclein, and Aβ_42_ levels in the CSF were best fit with a parabolic (not straight) line in PD, but not HC subjects. CSF IL-6 levels were best fit with a parabolic line in HC, but not PD

## Results

**Fig. 1:**
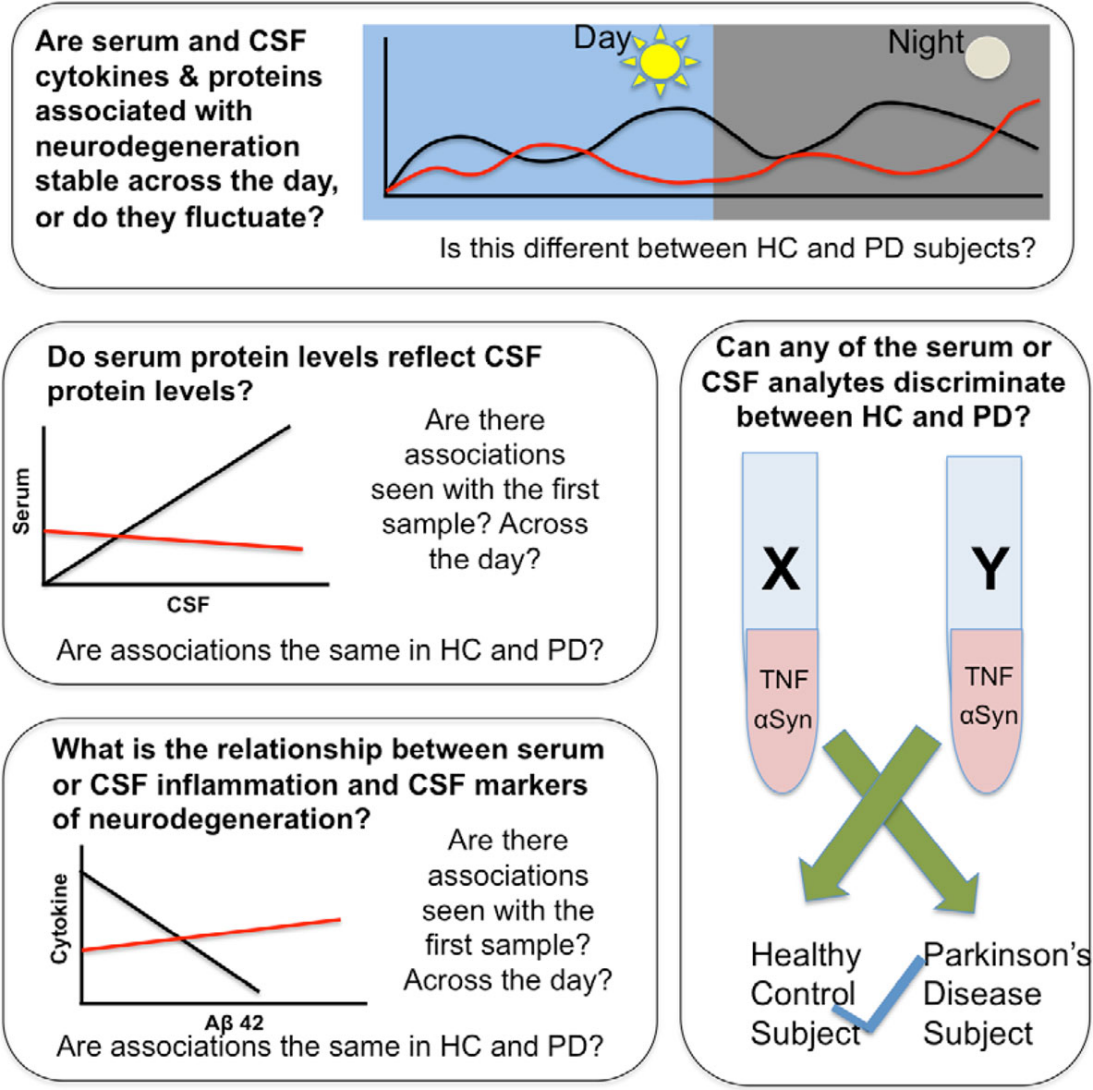
Key questions: inflammatory markers and neurodegeneratation biomarkers in PD and HC subjects across time. Our analysis addressed the following *a priori* specified questions aimed at establishing candidate inflammatory biomarkers to focus a larger cross-sectional or longitudinal study: Investigate the extent to which each analyte varied over time in serum and CSF, the extent of correlation between serum and CSF inflammatory factors, the extent of correlation between levels of known markers of neurodegeneration and serum and CSF inflammatory factors, and identification of analytes capable of discriminating between HC and PD subjects with high sensitivity and specificity

While multiple groups have reported alterations in inflammatory markers in the blood and CSF of individuals suffering from neurodegenerative diseases like PD [1], results are discordant [28]. To investigate the extent to which variability between individuals, sampling times, or diurnal patterns in specific inflammatory proteins in the blood or CSF contribute to the lack of consensus, and to generate hypotheses, we pursued a set of specific questions that required a complex set of comparisons of intra-individual and inter-group values (Fig. 1) to investigate the extent of association between inflammatory markers and other parameters of interest.

We measured a subset of inflammatory factors (i.e., IL-1β, IL-2, IL-6, IL-8, IL-4, IL-10, IL-12, IL-13, IFNγ, TNF, NGAL, and CRP) in the serum and CSF of PD and age-matched HC subjects. IL-1β, IL-2, IL-4, IL-10, IL-12, and IL-13 were not reliably above the lower limit of detection for the MSD assay and therefore were excluded from analyses. We also examined the relationship between the consistently detectable inflammatory factors and CSF levels of more established neurodegenerative disease-specific biomarkers, specifically α-synuclein, Aβ_40_, and Aβ_42_. Finally, we examined the relationship between serum and CSF factors and PD severity and duration.

### Serum IFNγ, IL-8, NGAL, and TNF and CSF IL-8, NGAL, and TNF levels were relatively stable across the day in most PD and HC individuals

Clinical heterogeneity and inter-individual variability are significant challenges to interpreting clinical data and critical determinants of statistical power. Thus, we first determined individual variability in inflammatory factors across 24 h. Regression analyses revealed marked variability across time in multiple inflammatory factors in both HC and PD subjects (Additional file 1). The most stable analytes across time in the majority of HC and PD subjects were serum IFNγ, IL-8, NGAL, and TNF and CSF IL-8, NGAL, and TNF. These inflammatory factors were stable in greater than two-thirds of subjects (Additional file 1). Serum and CSF NGAL were stable in 50% or more of both PD and HC subjects, indicating relative stability. While CSF CRP varied across time in slightly half of PD subjects (i.e., CRP levels increased over time in 58.3% of PD subjects), CSF CRP showed minimal variability in the majority of individuals in the HC population (i.e., CRP levels increased over time in only 33.3% of HC subjects). Serum IL-6, CSF IFNγ, and CSF IL-6 displayed an increase over the 24-h period in greater than two-thirds of HC subjects, whereas in PD subjects these analytes were mostly stable across time.

### Serum TNF, NGAL, and IFNγ are different at baseline in PD versus HC and remain relatively stable across the day

**Fig. 2:**
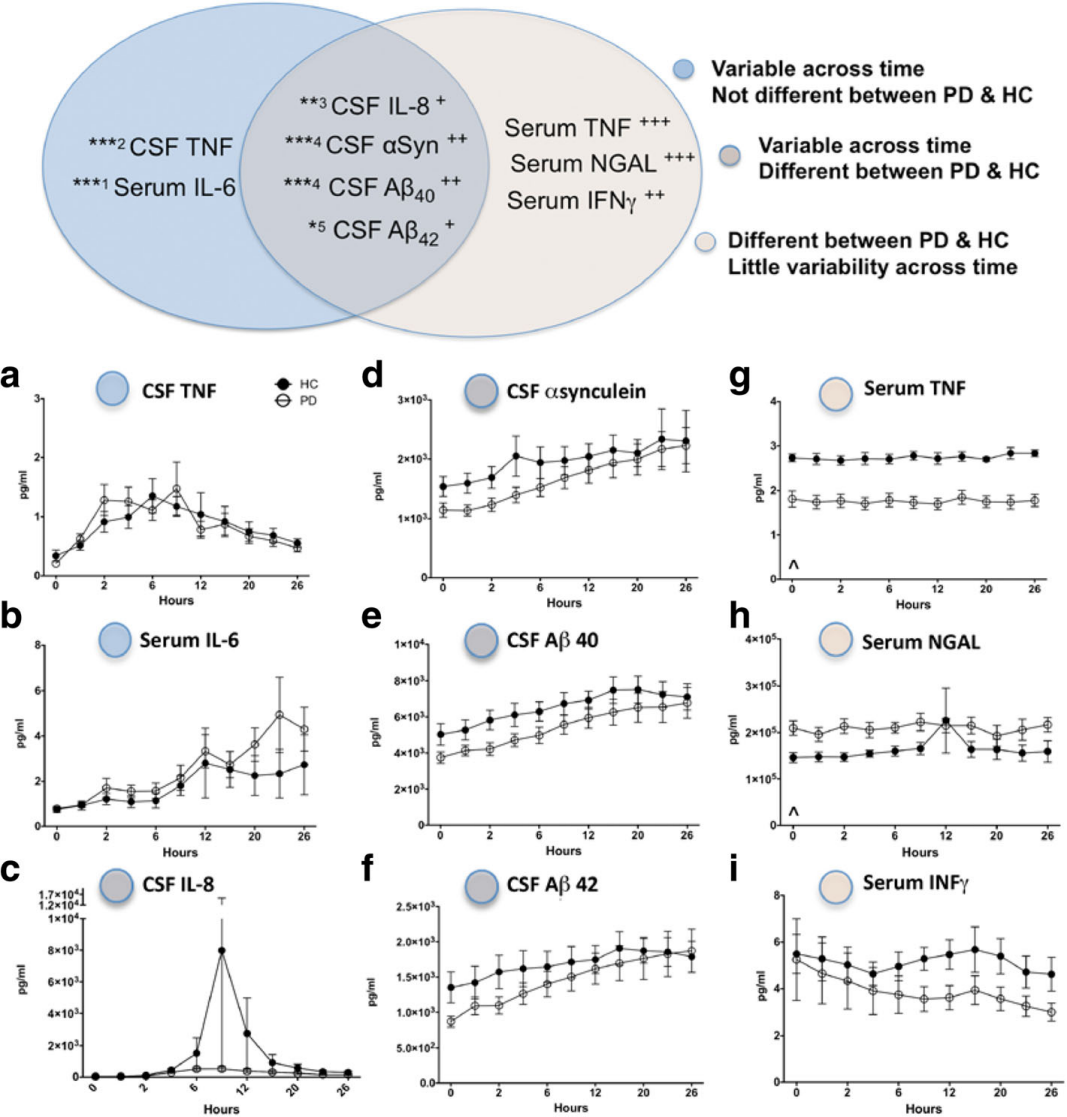
Serum and CSF profiles differ between PD and HC subjects and show distinct diurnal fluctuations. The *top diagram* summarizes significant results. When sampled across a 26-h period, protein levels of CSF TNF (**a**), and serum IL-6 (**b**) fluctuate significantly across the day (*F*_(10,154)_ = 4.66; *p* < 0.00001, and *F*_(10,160)_ = 3.12; *p* < 0.00001, respectively). CSF IL-8 (**c**), α-synuclein (**d**)**,** Aβ_40_ (**e**), and Aβ_42_ (**f**) are decreased in PD as compared to age-matched HC (*F*_(1,16)_ = 5.08; *p* = 0.04, *F*_(1,16)_ = 8.35 *p* = 0.01, *F*_(1,16)_ = 14.69; *p* = 0.001, and *F*_(1,16)_ = 4.61; *p* = 0.05, respectively), and vary across the day (*F*_(10,154)_ = 2.23; *p* = 0.01, *F*_(10,160)_ = 3.30; *p* < 0.001, *F*_(10,160)_ = 4.11; *p* < 0.0001, and *F*_(10,160)_ = 2.15; *p* = 0.02, respectively). Serum TNF (**g**) and serum IFNγ (**i**) are decreased in PD subjects compared to HC subjects (*F*_(1,16)_ = 208.58; *p* < 0.00001 and *F*_(1,16)_ = 8.49; *p* = 0.009, respectively), and serum NGAL (**h**) is increased in PD compared to HC subjects (*F*_(1,16)_ = 25.98; *p* < 0.00001). When measured at baseline (time 0 = 5:30 AM), serum TNF (**g**) was significantly lower in PD subjects as compared with HC subjects (*U*_(83, 88)_ = 10; *p* = 0.014), and serum NGAL (**h**) was significantly higher in PD subjects as compared with HC subjects (*U*_(31, 140)_ = 10; *p* = 0.014). There were no differences between PD and HC in any other analyte (see Table 2 for all data). *Indicates significant change in time, and + indicates significant difference between PD and HC. *** and ^+++^ indicate *p* < 0.0001, ** and ^++^ indicate *p* < 0.01, * and ^+^ indicate *p* ≤ 0.05, and ^ indicates a significant difference between PD and HC at time 0; *p* < 0.05. Superscript numbers indicate the sampling hour where significant changes occur

Despite finding significant within group variability and a relatively small sample size, we found significant differences between serum TNF and serum NGAL levels in PD versus HC at baseline (time 0; Fig. 2). PD subjects had lower levels of serum TNF (Fig. 2g); *U*_(83, 88)_ = 10; *p* = 0.01) and higher levels of serum NGAL (*h*; *U*_(31, 140)_ = 10; *p* = 0.01) at baseline than age-matched HC subjects (see Additional file 2). A repeated measures two-way analysis of variance (ANOVA) by time and disease state for each analyte (Fig. 2 and Additional file 3) revealed that certain serum and CSF analytes changed over time but not in a disease-specific manner (CSF TNF; Fig. 2a and serum IL-6; Fig. 2b, left side of Fig. 2 diagram), while others were different between PD and HCs but not stable across time (CSF IL-8; Fig. 2c, CSF α-synuclein; Fig. 2d, CSF Aβ40; Fig. 2e, and CSF Aβ42; Fig. 2f, middle in Fig. 2 diagram). However, serum TNF (Fig. 2g), serum NGAL (Fig. 2h), and serum IFNγ (Fig. 2i) were stable over the 24-h period and were different between PD and HC groups. Serum TNF (Fig. 2g) and serum IFNγ (Fig. 2i) were lower in PD subjects compared to HC subjects (*F*_(1,16)_ = 208.58; *p* < 0.0001, and *F*_(1,16)_ = 8.49; *p* = 0.009, respectively), and serum NGAL (Fig. 2h) was higher in PD compared to HCs (*F*_(1,16)_ = 25.98; *p* < 0.0001). All three analytes showed minimal variability across time (*F*_(10,160)_ = 0.11; *p* = 1.00, *F*_(10,160)_ = 0.38; *p* = 0.95, and *F*_(10,160)_ = 0.73; *p* = 0.69, respectively). CSF α-synuclein, Aβ_40_, and Aβ_42_ were significantly lower in PD patients as compared with HC subjects (*F*_(1,6)_ = 8.35; *p* = 0.01, *F*_(1,6)_ = 14.69; *p* = 0.001, and *F*_(1,6)_ = 4.61; *p* = 0.05, respectively). Depending on time of day, these CSF markers were different in PD versus HC subject groups. There were no differences between PD and HC across time in any other analytes.

### Inflammatory proteins in CSF display greater fluctuation in PD versus HC across the day

We next determined whether inflammatory analytes, α-synuclein, and/or Aβ_40_ and Aβ_42_ levels fluctuate across the day by examining quadratic trends for each analyte across time (Table 1). The pattern of CSF TNF (*p* = 0.02), CRP (*p* = 0.03), IL-8 (*p* = 0.02), and Aβ_42_ (*p* = 0.006) levels across the day were best fit by a parabolic (not linear) relationship in PD subjects, but not in HCs (*p* = 0.07, *p* = 0.25, *p* = 0.31, *p* = 0.06, respectively), indicating that the levels of these CNS proteins rise and fall across the day more in PD subjects versus HC. CSF IL-6 levels rise and fall across in the day in HC subjects (*p* = 0.01), but not in PD (*p* = 0.12), and CSF Aβ_40_ levels across the day were best fit by a quadratic trend in both PD (*p* = 0.02) and HC (*p* = 0.004) subjects.

### PD and HC subjects displayed different relationships between serum and CSF inflammation across the day

Next, we investigated the extent to which levels of an inflammatory marker in serum correlated with its levels in the CSF (Additional file 4). Serum CRP and CSF CRP significantly co-varied across time in one group but not the other. Serum IL-6 and CSF IL-6 were significantly related in both groups, and the relationships were different in PD and HC subjects. Serum NGAL and CSF NGAL displayed a significant association that was not different between PD and HC subjects. Serum IFNγ, serum IL-8, and serum TNF did not covary with CSF analytes but were different between the two groups across time (see Additional file 4 for statistics). These data indicate that CSF and serum levels of IL-6 and CRP display significant correlations across a 24-h period and that they do so in a unique way in HC and PD subjects.

### CSF and serum CRP positively covary in PD and HC subjects at baseline

Next, we examined the relationship between serum and CSF inflammatory factors using the samples from the first collection period (time 0). We found no significant correlation between serum and CSF levels of any factor except CRP (Additional file 6a). Serum and CSF CRP significantly correlated in HC (slope = 325 ± 86.9, *F*_(1, 4)_ = 14.0; *p* = 0.02) and in PD (slope = 415.5 ± 26.6, *F*_(1, 10)_ = 243.8; *p* < 0.0001), but the slopes did not differ between HC and PD subjects (*t*(16) = 1.28; *p* = 0.22) (a). Serum and CSF NGAL (b), TNF (c), IL-6 (d), IL-8 (e), and IFNγ (f) did not correlate at baseline, indicating that serum and CSF levels of these factors do not reflect one another (Additional file 6).

### PD and HC subjects displayed different relationships between biofluid inflammation and CSF α-synuclein, Aβ_40_, and Aβ_42_ proteins across the day and at baseline

Next, we investigated the relationship between levels of inflammatory markers and α-synuclein, Aβ_40_, and Aβ_42_, which are frequently assayed as potential biomarkers of neurodegenerative disease (Additional file 5). Interestingly, with respect to CSF α-synuclein, HC and PD subjects displayed different relationships between this biomarker and serum IFNγ, serum NGAL, serum CRP, CSF TNF, and CSF CRP. With respect to CSF Aβ_40_, HC and PD subjects displayed different relationships between this biomarker and serum IFNγ, serum NGAL, serum CRP, serum IL-8, CSF TNF, CSF NGAL, CSF CRP, and CSF IL-8. Finally, with respect to Aβ_42_, HC and PD subjects displayed different relationships between this biomarker and serum IFNγ, serum CRP, CSF TNF, CSF NGAL, CSF CRP, CSF IL-6, and CSF IL-8. (see Additional file 5 for statistics).

**Fig. 3:**
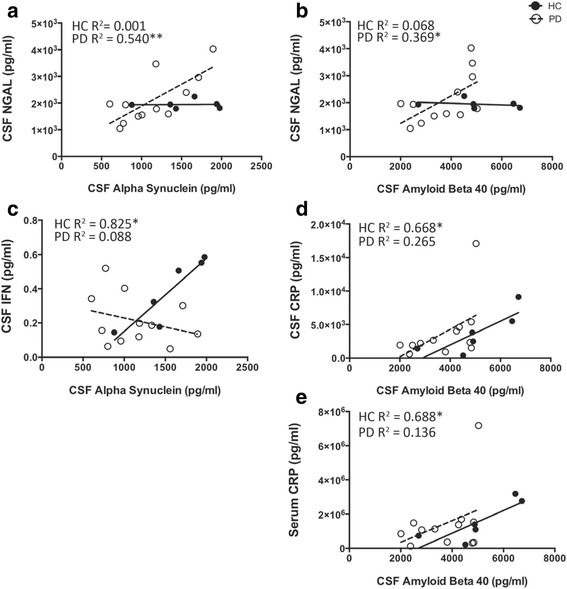
At time 0, CSF NGAL, IFNγ, CRP, and serum CRP disease-dependently covary with Aβs. PD subjects demonstrate a relationship between CSF NGAL and CSF α-synuclein (**a**; *F*(1,10) = 11.73; *p* = 0.007) as well as CSF NGAL and CSF Aβ40 (**b**; *F*(1,10) = 5.86; *p* = 0.04), while HC subjects do not. HC subjects demonstrate a relationship between CSF IFNγ and CSF α-synuclein (**c**; *F*(1,4) = 18.88; *p* = 0.01), CSF CRP and Aβ40 (**d**; *F*(1,4) = 8.03; *p* = 0.05), and serum CRP and Aβ40 (**e**; *F*(1,4) = 8.80; *p* = 0.04), while PD subjects do not (see Additional file 7 for all correlation data). * indicates *p* < 0.05 and ** indicates *p* < 0.01

Next, we examined the relationship between CSF α-synuclein, Aβ40, and Aβ42 levels with serum and CSF inflammatory factors analyzed at baseline (time 0). At time 0, CSF NGAL, CSF IFNγ, CSF CRP, and serum CRP co-varied with α-synuclein, Aβ_40_, and Aβ_42_ in a disease-dependent manner (Fig. 3): PD subjects demonstrated a relationship between CSF NGAL and CSF α-synuclein (Fig. 3a) as well as CSF NGAL and CSF Aβ_40_ (b), while HC subjects did not. HC subjects demonstrated a relationship between CSF IFNγ and CSF α-synuclein (c), CSF CRP and CSF Aβ_40_ (d), and serum CRP and CSF Aβ_40_ (e), while PD subjects did not (Fig. 3; Additional file 7 for statistics).

### CSF IFNγ and serum IL-8 positively correlate with some clinical measures in PD subjects, and serum TNF and CSF α-synuclein correctly categorize individuals into PD or HC groups with high specificity and sensitivity

**Fig. 4:**
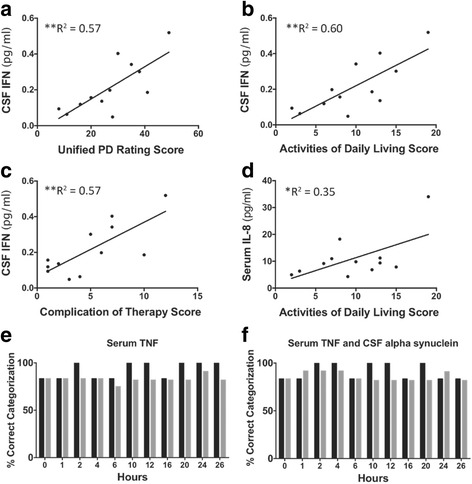
Inflammation correlates with UPDRS and predicts disease phenotype with sensitivity and specificity across the day. When measured at baseline (time 0 = 5:30 AM), CSF IFNγ positively correlates with the UPDRS (**a**; *p* = 0.004), as well as the Activities of Daily Living Score component of the UPDRS (**b**; *p* = 0.003) and the Complication of Therapy Score (**c**; *p* = 0.005). Serum IL-8 positively correlated with the Activities of Daily Living Score (**d**; *p* = 0.04). There were no other significant correlations between inflammation and disease severity or number of years with disease. Serum TNF alone (**e**) and serum TNF with CSF α-synuclein (**f**) demonstrated ascending accuracy and precision in discriminating between HC and PD subjects when analyzed with linear discriminant analyses (LDA; see Additional file 9: Figure S2 for all LDA factors considered)

Next, we analyzed relationships between serum and CSF inflammation and clinical measures of disease state as determined by the UPDRS, its components (mentation, behavior, and mood score, activities of daily living score, motor examination score, and complication of therapy score), and disease duration (years since PD diagnosis; Fig. 4 and Additional file 8 for statistics). Interestingly, levels of CSF IFNγ increased as the UPDRS increased (Fig. 4a). When UPDRS component scores were examined separately, CSF IFNγ levels increased as the activities of daily living (ADL) score (Fig. 4b) and the complication of therapy score (Fig. 4c) increased, indicating that the highest levels of CSF IFNγ are found in PD subjects with the most disruption of daily living activities and the most complication experienced due to therapeutic treatment. Serum IL-8 levels demonstrated a significant positive relationship with the ADL score (Fig. 4d), such that subjects with the highest levels of serum IL-8 have the most disruption in their daily activities due to PD. There were no other significant correlations noted between PD severity or disease duration and inflammation in the serum or CSF. There were no relationships noted between serum or CSF inflammatory markers and years with PD or disease severity or duration and CSF neurodegenerative markers (i.e., α-synuclein, Aβ_40_, and Aβ_42_; data not shown).

Given that inflammatory status results from a convergence of multiple variables and factors, we used linear discriminant analysis (LDA) to determine if any particular analyte or a particular set of analytes allowed for correct assignment of group membership (with all sampling time points considered). LDA revealed that inflammation and a neurodegenerative disease marker correctly assign individuals to HC or PD groups. Indeed, serum TNF alone misclassified only 17% of PD subjects into the HC group and only 17% of HC subjects (Fig. 4e). Serum TNF with CSF α-synuclein together correctly categorized > 75% of both PD and HC groups across all time points (Fig. 4f; 82% sensitivity and 83% specificity) which was the most accurate of all parameter combinations tested.

Serum and CSF IL-6 alone incorrectly assign 100% of HC individuals into the PD group (Additional file 9). While serum and CSF levels of IL-6 correctly categorized at least 75% of individuals into the PD group across time (Additional file 9a), these two factors were not sufficient to discriminate between HC and age-matched PD subjects. Similar results were obtained when only serum IL-6 was considered (Additional file 9b for all data).

## Discussion

Several studies have investigated the extent to which Parkinson's disease (PD) pathophysiology is associated with increased inflammatory status. However, due to conflicting results [28], there is no agreement on which inflammatory proteins hold promise as potential biomarkers to stage or monitor disease progression, and a clear picture of biofluid inflammation in PD is stymied by a complete lack of knowledge of the extent of diurnal fluctuations in inflammatory proteins in peripheral (serum, plasma) and central (CSF) compartments in both healthy control (HC) subjects and PD patients. Here, we analyzed the levels of six inflammatory proteins (i.e., IL-6, IL-8, IFNγ, TNF, NGAL, and CRP) concurrently across a 26-h sampling period in the serum and CSF of PD (*n* = 12) and age-matched HC subjects (*n* = 6). The key findings are summarized in Table 2.

**Table 2:** Summary of findings. Serum IFNγ, serum NGAL, serum TNF, and serum CRP, CSF NGAL, CSF TNF, CSF IFNγ, and CSF CRP display several characteristics of candidate biomarkers

Biomarker characteristic	Analyte
Diurnal stability in most PD and HC individuals (Additional file 1; top)	Serum IFNγ, IL-8, and TNF CSF IL-8 and TNF
Diurnal stability and different between PD and HC (Additional file 1; bottom, Fig. 2, Additional file 2, Additional file 3, and Table 1)	Serum IFNγ, NGAL, and TNF
Different relationship between CSF and serum levels across the day in PD and HC (Additional file 4)	Serum and CSF CRP and IL-6
CSF and serum levels covary at time 0 in both PD and HC subjects (Additional file 6)	CRP
Covary with α syn and Aβ peptides across the day, and PD and HC subjects have different diurnal relationships (Additional file 5)	Serum IFNγ and CRP CSF TNF and CRP
Different relationship with α syn and Aβ peptides between PD and HC at time 0 (Fig. 3 and Additional file 7)	Serum CRP CSF IFNγ, NGAL, and CRP
Covary with UPDRS or components (Fig. 4 and Additional file 8)	Serum IL-8 CSF IFNγ
Correctly categorizes ≥ 75% of individuals into appropriate group (PD or HC; Additional file 9)	Serum TNF alone (75% +) Serum TNF with CSF α syn (80% +)

We stress that the sample size of this study is very small and designed to generate new hypotheses and inform a larger study. While age and BMI were controlled for (Additional file 10), and inclusion/exclusion criteria were strictly adhered to for recruitment of subjects (Additional file 11), some factors that may influence cytokine profiles were not considered. For example, PD subjects taking a stable dose of PD medication (amantadine, dopamine agonists, L-DOPA, and/or MAO-B inhibitors) for 4 weeks prior were included, and drug dose and drug type were not controlled. Dopaminergic drugs increase inflammation in PD and PD models [45–48], and increased inflammation after long-term L-DOPA treatment may contribute to the development of dyskinesia [46, 47]. However, there is some evidence that DA drugs decrease inflammation [49] or do not change circulating inflammation at all [50]. Our concerns about the potential confounding effects of dopaminergic therapy are lessened given that dopaminergic drugs did not skew inflammatory levels up or down across the board. Despite the fact that not all patients were on the same kinds of medication, which is usually the case in the clinic, we were able to find PD-specific attributes to some of the analytes. These and other factors could influence inflammation [51, 52], and should be taken into account in future studies.

While we found significant temporal variability in both PD and HC groups across the day in many serum and CSF inflammatory factors, serum IFNγ displayed stability across the day in 83% of HC individuals and 92% of PD individuals, and both serum and CSF TNF displayed stability across the day in 100% of HCs and 92% of PD subjects. These data suggest NGAL and particularly TNF in the serum may be good candidate biomarkers to pursue as indicators of inflammatory state in cross-sectional studies where it may not be possible to adhere to an exact sampling time. Differences were noted between PD and HC groups in serum TNF and NGAL at time 0 with TNF being significantly decreased, and NGAL being significantly increased in PD. While serum IFNγ levels are more variable within groups than serum TNF or NGAL, significant differences in serum IFNγ were noted between HC and PD groups when all time points are considered, with IFNγ being significantly lower in PD subjects as compared with HC subjects. However, differences in serum IFNγ between PD and HC subjects were not noted at baseline (time 0), indicating that multiple sampling points may be required. However, repeated sampling protocols increase intra-subject variability of CSF Aβ protein levels [53], highlighting the importance of choosing a relatively stable biomarker.

Identification of TNF and IFNγ as potential candidate biomarkers is noteworthy given that both of these cytokines have been strongly implicated in degeneration of nigral dopaminergic neurons and basal ganglia pathologies in pre-clinical and post mortem studies [54–63]. Levels of serum TNF have been reported to be increased in PD subjects compared with age-matched HC subjects [33–35, 37], but decreased serum TNF levels [38] as well as no difference in serum TNF levels [64] have been reported in PD versus HC subjects. As our data indicate stability in the levels of serum TNF across the day both individually, and within groups, we likely rule out that diurnal variability accounts for this disparity in the TNF literature. In a recent meta-analysis of inflammatory serum levels in PD subjects and HCs, Qin and colleagues [28] determined that of the 25 studies considered in the analysis, 9 demonstrated an increase of serum TNF levels in PD. However, age was found to be a confounding factor. Like serum TNF, serum IFNγ has also been demonstrated to be increased [33] and decreased [34] in PD subjects compared with HCs. Here, we demonstrate that when all sampling time points are considered, there is a discernable decrease in serum IFNγ in PD subjects compared with HCs. These data suggest that sampling time (and/or other circumstances of collection) could account for IFNγ variability reported in the literature, as we found no difference between HC and PD subjects in serum IFNγ at time 0, and a moderate, though insignificant, degree of variability across the 24-h period in both HC and PD groups. Standardizing sampling paradigms (e.g., number of samples, volume of each sample, total sample volume) and differences between CSF collection methods (e.g., gravity drip, syringe draw, peristaltic pump in a closed system as used here) would likely reconcile some inconsistencies in the literature [53].

Together, our results strongly suggest that serum TNF and serum NGAL are the most promising candidate inflammatory biomarkers because they remain relatively invariant throughout a 24-h period in the majority of subjects and because their levels are significantly different between PD and HC groups both at time 0 and across the day. To our knowledge, this is the first study to investigate NGAL protein levels in blood or CSF of PD subjects. NGAL (also known as lipocalin 2, 24p3, uterocalin, and siderocalin) is an acute phase protein [65] involved in innate host defense against bacteria [66] that is both upstream and downstream of TNF signaling, and sensitizes cortical neurons to β-amyloid toxicity [67]. Several studies have identified increases in NGAL in subjects with familial amyloid polyneuropathy [68] and more recently in patients with late-life depression [69-71], Down’s syndrome with dementia [72], and Alzheimer’s disease with depression [73]. Finally, CSF NGAL holds great potential as a novel companion inflammatory biomarker in PD because it is easy to measure, it is stable across time, and it is part of a signaling network with TNF [67].

We next demonstrated that CSF TNF, CRP, IL-8, and Aβ_42_ levels fit a quadratic equation indicating a parabola shape when graphed across the day (i.e., significantly changed from time 0 levels and then significantly changed back toward time 0 levels) in PD subjects but not HCs. CSF IL-6 levels rise and fall across in the day in HC subjects, but not PD, and CSF Aβ_40_ levels across the day were best fit by a quadratic trend in both PD and HC subjects. CSF TNF has the clearest pattern of change across the day, increasing from below 1 pg/mL at 5:30 AM to around 2 pg/mL at around 11:30 AM and finally back down to below 1 pg/mL at 5:30 AM the next day. However, there was no difference in CSF TNF levels between PD and HC subjects at any time point, and the lack of significant quadratic trend in HC CSF TNF is likely due to a small sample size. Although few studies have sampled biofluids across the day to determine diurnal inflammatory patterns, one study in adult insomnia patients and age-matched HCs (~ 27–31 years old) found disturbed rhythms in plasma IL-6 and increased plasma TNF (but no diurnal rhythm) in insomniacs, when measured every 30 min across 24 h [74]. Although we have no information on the extent of sleep disruption in subjects participating in this study, we noted a decrease in serum TNF with disease, we similarly noted stable TNF levels across the day in PD and HC subjects and found similar levels of IL-6 and TNF in our ~ 50-year old subjects (~ 3–6 pg/ml and ~ 2–3 pg/ml, respectively). While we did not find significant rhythmicity in serum IL-6 across the day, this could be due to our small sample size as the plasma IL-6 pattern noted by Vgontzas and colleagues [74] bears striking resemblance to what we noted for serum IL-6 with a more robust increase and then apparent decrease across the day in PD subjects and insomniacs versus HC subjects whom displayed a relatively flat profile across the day in both studies [74]. Additionally, our data confirm previous reports demonstrating rhythmicity in CSF Aβ_40_ and Aβ_42_ across the day [75]. Together, these data suggest that there is more variability in central inflammation across the day in PD subjects as compared with HCs. Cytokines and other proteins (including cortisol) are significantly increased after knee surgery, and rise and fall more in the CSF than in the serum, indicating that increased fluctuation of CSF proteins as compared to serum levels is not uncommon [76]. One reason could be due to the relatively low dilution of cytokines by CSF versus the much higher volume of the blood circulatory system, and Bromander et al. speculate that the greater fluctuation seen in CSF versus serum could be because the inflammatory systems of the brain and the periphery are regulated separately, and suggest that CSF cytokine fluctuation may indicate BBB disruption, a characteristic known to be associated with neurodegenerative disease [77].

Though serum and CSF inflammatory factors have been suggested to reflect one another, there are conflicting reports [28]. Therefore, we investigated the relationship between serum and CSF levels of all detectable analytes in HC and PD subjects across the day and found that levels of IL-6 and CRP covary in serum and CSF across the day, and they do so in a disease-dependent manner. Surprisingly, we found that of all analytes evaluated at time 0, CRP is the one inflammatory factor in serum that reflects levels in CSF (although concentration ranges are an order of magnitude lower in CSF). While we demonstrated no difference between serum or CSF CRP between HC and PD subjects, CRP has been reported to be associated with increased risk of death and indicative of life expectancy in PD subjects [78]. These discrepancies could be accounted for by individual variability, as there was substantial within-subject variability in serum and CSF CRP across the 24-h sampling period. Interestingly, while CRP levels were variable across the day in the serum and CSF of the majority of PD subjects, CSF CRP levels were stable in the majority of HC subjects, indicating that diurnal patterns of CSF CRP may be disrupted in association with PD. These important findings suggest that blood analysis of CRP could feasibly be used to probe neuroinflammation to inform inflamm-aging [79–81] or disease status without a need for the more invasive lumbar catheter puncture for CSF collection. Additionally, mechanism-based hypotheses about neuroinflammatory status may be gleaned from analyzing existing data on serum CRP levels in PD subjects.

Alpha (α)-synuclein, Amyloid-beta-40 (Aβ_40_), and Aβ_42_ are proteins currently under intense investigation as potential biomarkers of neurodegenerative disease. Levels of inflammatory factors in the brain are likely to be changing prior to frank neuronal death because microglia, the innate immune cells in the brain produce cytokines when activated in response to aggregated proteins [1, 82]. A wealth of evidence indicates that toxic oligomers of α-synuclein and Aβ peptides trigger inflammatory responses in vitro and in vivo and compromise neuronal health and survival [83–85] and that brain inflammation, in turn, increases aggregation of those oligomers [25, 86–88]. Our data demonstrate that, across time, serum IFNγ, serum CRP, CSF TNF, and CSF CRP covary with all three biomarkers of neurodegenerative disease (α-synuclein, Aβ_40_, and Aβ_42_) in a disease-dependent manner. These exciting and novel data suggest that serum and CSF inflammation may be associated with abnormalities in CSF levels of α-synuclein, Aβ_40_, and Aβ_42_. Interestingly, all significant relationships between CSF and serum inflammation and CSF toxic peptide levels at time 0 are positive such that as inflammation in CSF or periphery increases, so do CSF α-synuclein and Aβ peptides, providing additional evidence that inflammation and toxic oligomer species may be part of a feed-forward cycle of protein aggregation-neuroinflammation.

The Unified Parkinson’s Disease Rating Scale (UPDRS) is comprised of several components including the mentation, behavior, and mood score, the activities of daily living score (ADL), the motor evaluation score, and the complications of therapy score. Our data indicate that serum IL-8 levels were significantly and positively associated with the ADL scores component of the UPDRS. As serum IL-8 was stable across the day in the majority of PD individuals, this factor may be of interest in future longitudinal studies to determine whether it is associated with disease severity irrespective of disease duration or time of day. Consistent with this idea, the association of IL-8 with clinical severity was also reported in a recent analysis of human serum in a multi-center cohort of 142 subjects with familial PD arising from leucine rich repeat kinase 2 (LRRK2) mutations where high levels of IL-8, MCP-1, and CCL4 were associated with the presence of a specific clinical subtype that is characterized by a broad and more severely affected spectrum of motor and non-motor symptoms [89]. With regards to inflammatory markers in the CSF, IFNγ levels were higher in PD individuals with higher UPDRS total scores, higher activities of daily living (ADL) scores, and in PD individuals that have more complications from therapeutic treatment, suggesting that IFNγ levels may be of particular interest in disease staging and monitoring. IFNγ regulates the expression of major histocompatibility complex II (MHCII) on monocytes, microglia, and macrophages [90]. A single nucleotide polymorphism located in the first intron of the MHCII Human Leukocyte Antigen (HLA)-DRA gene was found to be significantly associated with sporadic PD in a recent genome wide association study [91], indicating that IFNγ may contribute to disease severity by affecting antigen presentation and the resulting inflammatory response. Finally, increased expression of IFNγ in the CNS driven by a viral vector in mouse brain resulted in basal ganglia calcification and nigrostriatal degeneration, reminiscent of human idiopathic basal ganglia calcification (IBGC) [63].

Given that PD is an extremely heterogeneous disease with differing rates of progression [35, 92], our results demonstrating that inflammation did not increase with years since PD diagnosis are not entirely surprising. Indeed, these findings are in line with imaging data demonstrating increased inflammation in the pons, basal ganglia, striatum, and cortex of PD subjects irrespective of disease duration [13], lending credence to the hypothesis that changes in inflammation likely occur early in disease and remain present throughout the course of disease. One additional potential reason for the finding that these inflammatory markers positively correlate with some measures of PD severity but not duration is the fact that the biofluids analyzed in this study were taken from a MJFF repository originating from an experimental medicine study that selected subjects able to tolerate the continuous CSF procedures. Therefore, it is a subset of the PD population (usually younger) and therefore with shorter duration of illness. However, severity of PD in this population might well be related to individuals with more aggressive deterioration and therefore linked to inflammatory biomarkers. The potential value of inflammatory biomarker assessment is in its predictive ability in identifying who is likely to have a faster rate of decline despite years with disease. The key to resolving this issue is to replicate the findings in a larger cohort of subjects.

We used linear discriminant analysis (LDA) to determine if any particular analyte or set of analytes allowed for correct assignment of any one sample to PD or HC group membership across time. To our surprise, there was a relatively minimal misclassification when only serum TNF levels were considered; serum TNF alone misclassified, at most, only 25% of PD subjects (3 of 12) into the HC group, and 33% of HC (2 of 6) subjects into the PD group across all time points. When serum TNF and CSF α-synuclein levels are considered together, ≥ 82% sensitivity and 83% specificity were achieved in both HC and PD groups across all time points; CSF tau and Aβ demonstrate comparable sensitivity (but less specificity) as diagnostic tools for Alzheimer’s disease [93]. Together, these analyses indicate that, at a minimum, TNF measured in the serum and α-synuclein measured in the CSF have high potential utility for sensitive and specific detection of PD state.

## Conclusions

In summary, our data indicate that serum and CSF NGAL, TNF, IFNγ, and CRP, and serum IFNγ are promising candidate biomarkers of inflammation. Importantly, we emphasize that the sample size in our study was very small and confirmatory studies will be needed in a larger cohort of subjects to validate these findings using current biorepositories with banked serum and CSF, details regarding the employed collection protocol, and good clinical history data for each subject (such as in the PPMI, Precept/PROBE and HBS cohorts [94] as well as in the DeNoPa cohort [44]), including information about autoimmune and chronic systemic disease (diabetes, obesity, cardiovascular disease, etc.) and other comorbidities that could influence the levels of inflammatory proteins in their biofluids. In the future, we propose that a similar 24-h collection study should be performed in subjects experiencing prodromal pre-motor symptoms of PD to investigate the extent to which the factors we identified could be used to aid in earlier diagnosis of individuals at risk and to monitor their disease trajectory, and hope that the data reported in this small study will generate hypotheses regarding potential inflammatory profiles in patients with neurodegenerative disease.

## Additional files

Additional file 1: Regression analysis of each analyte by individual and means and SEM by analyte across time. (PDF 1526 kb)

Additional file 2: Serum TNF and NGAL are significantly different between PD and HC at time 0. (PDF 561 kb)

Additional file 3: Serum TNF, IFNγ, and NGAL are different between PD and HC irrespective of time. (PDF 1693 kb)

Additional file 4: PD and HC subjects have different relationships between serum and CSF inflammation. (PDF 801 kb)

Additional file 5: Summary of relationships between inflammation and Aβs and α-synuclein in PD and HC. (PDF 2346 kb)

Additional file 6: Serum and CSF CRP levels are positively correlated in HC and PD at baseline. The relationship between serum and CSF inflammatory factors was analyzed using samples from the first collection period (baseline; time 0). There was no significant correlation between serum and CSF levels of any factor except CRP (a). Serum and CSF CRP significantly correlate in HC (slope = 325 ± 86.9, *F*_(1, 4)_ = 14.0; *p* = 0.02) and in PD (slope = 415.5 ± 26.6, *F*_(1, 10)_ = 243.8; *p* < 0.0001), but the slopes do not differ between HC and PD (*t*(16) = 1.28; *p* = 0.22). Serum and CSF NGAL (HC; slope = 66.57 ± 64.63, *R* ^2^ = 0.17, *F*_(1,4)_ = 0.80; *p* = 0.42 and PD; slope = 3.44 ± 18.28, *R* ^2^ = 0.003, *F*_(1,10)_ = 0.04; *p* = 0.85) (b), TNF (HC; slope = −0.33 ± 0.40, *R* ^2^ = 0.15, *F*_(1,4)_ = 0.68; *p* = 0.46 and PD; slope = 2.15 ± 2.13, *R* ^2^ = 0.09, *F*_(1,10)_ = 1.02; *p* = 0.34) (c), IL-6 (HC; slope = −0.05 ± 0.09, *R* ^2^ = 0.07, *F*_(1,4)_ = 0.32; *p* = 0.60 and PD; slope = −0.03 ± 0.11, R^2^ = 0.009, *F*_(1,10)_ = 0.09; *p* = 0.77) (d), IL-8 (HC; slope = 0.20 ± 0.17, *R* ^2^ = 0.25, *F*_(1,4)_ = 1.31; *p* = 0.32 and PD; slope = 0.06 ± 0.22, *R* ^2^ = 0.01, *F*_(1,10)_ = 0.08; *p* = 0.79) (e), and IFNγ (HC; slope = −5.64 ± 4.78, *R* ^2^ = 0.28, *F*_(1,4)_ = 1.59; *p* = 0.28 and PD; slope = 9.42 ± 12.77, *R* ^2^ = 0.05, *F*_(1,10)_ = 0.54; *p* = 0.48) (f) do not correlate at baseline. (PDF 74 kb)

Additional file 7: Baseline CSF α-synuclein and Aβs and CSF and serum inflammatory proteins between PD and HCs. (PDF 1977 kb)

Additional file 8: CSF IFNγ and serum IL-8 positively correlated with UPDRS components at baseline in PD subjects. (PDF 563 kb)

Additional file 9: Linear discriminant analysis reveals sensitivity and selectivity of analytes in discriminating between HC and PD. Serum and CSF levels of IL-6 (a), serum IL-6 (b). Serum and CSF IL-8, TNF, NGAL, and IFNγ, and CSF α-synuclein, Aβ40 and Aβ42 (d), serum IL-8, TNF, NGAL, IFNγ, and CSF α-synuclein, Aβ40, and Aβ42 (e), serum and CSF IL-8, TNF, NGAL, IFNγ, and CSF Aβ40 (f), serum and CSF IL-8, TNF, NGAL, IFNγ, and CSF Aβ42 (g), serum and CSF IL-8, TNF, NGAL, IFNγ, and CSF α-synuclein (h), serum and CSF IL-8, TNF, NGAL, IFNγ (i), serum IL-8, TNF, NGAL, IFNγ, and CSF Aβ42 (j), serum IL-8, TNF, NGAL, IFNγ, and CSF α-synuclein (k), serum IL-8, TNF, NGAL, IFNγ, and CSF Aβ40 (l), serum and CSF TNF, NGAL, IFNγ, and CSF α-synuclein, Aβ40, and Aβ42 (m), serum TNF, NGAL, IFNγ, and CSF α-synuclein, and Aβ42 (n), serum TNF, NGAL, and IFNγ (o), serum and CSF TNF, NGAL, and IFNγ (p), serum TNF, NGAL, and IFNγ and CSF α-synuclein, Aβ40, and Aβ42 (q), serum TNF and CSF α-synuclein, and Aβ42 (r), serum TNF and CSF Aβ42 (s), serum IL-8, TNF, NGAL, and IFNγ (t). The *x* axis is the percentage of correct categorization into a group, and the red line is set at the lowest percentage of correct categorizations for the factor or factors considered. The *y* axis is the collection hour. (PDF 239 kb)

Additional file 10: Subject demographics. (PDF 411 kb)

Additional file 11: Inclusion and exclusion criteria summary. (PDF 1759 kb)

## Abbreviations

ADLS: Activities of daily living score
ANCOVA: Analysis of covariance
ANOVA: Analysis of variance
Aβ: Amyloid β
Aβ_40_: β-Amyloid 1–40
Aβ_42_: β-Amyloid 1–42
BCA: Bicinchoninic acid assay
BMI: Body mass index
CCL4: Chemokine (C-C motif) ligand 4
CNS: Central nervous system
CRP: C-reactive protein
CSF: Cerebrospinal fluid
CV: Coefficient of variation
DA: Dopamine
ECG: Electrocardiogram
ECL: Electrochemiluminescent
ELISA: Enzyme-linked immunosorbent assay
H&Y: Hoehn and Yahr
HC: Age-matched healthy controls
HIV: Human immunodeficiency virus
HLA-DRA: Human leukocyte antigen - DR α chain
IBGC: Idiopathic basal ganglia calcification
IFNγ: Interferon γ
IL: Interleukin
LDA: Linear discriminant analysis
L-DOPA: L-3,4-dihydroxyphenylalanine
LRRK2: Leucine rich repeat kinase 2
MAO-B: Monoamine oxidase B
MCP-1: Monocyte chemoattractant protein-1
MHCII: Major histocompatibility complex II
MJFF: Michael J. Fox Foundation
MSD: ELISA Meso Scale Discovery
NFkB: Nuclear factor kappa B
NGAL: Neutrophil gelatinase-associated lipocalin
PD: Parkinson’s disease
PI: Primary investigator
RBC: Red blood cell
SEM: Standard error of the mean
TNF: Tumor necrosis factor
UPDRS: Unified Parkinson’s Disease Rating Scale
αSyn: α-Synuclein
