# Supplementary material for: Candidate inflammatory biomarkers display unique relationships with alpha-synuclein and correlate with measures of disease severity in subjects with Parkinson’s disease
Source: J Neuroinflammation. 2017 Aug 18;14:164. doi: 10.1186/s12974-017-0935-1 (PMC5563061; doi:10.1186/s12974-017-0935-1)
Supplement: Supplementary file 1 — Regression analysis of each analyte by individual and means and SEM by analyte across time. (PDF 1526 kb) [file 12974_2017_935_MOESM1_ESM.pdf]

Supplementary Table 1. Regression analysis of each serum and CSF analyte across time for each individual (within subjects comparison across time; top) and the means and standard error of the mean for each analyte across the day (bottom).

Analyte	Serum						CSF								
	CRP	IFNγ	IL-6	IL-8	NGAL	TNF	CRP	IFNγ	IL-6	IL-8	NGAL	TNF	αSyn	Aβ40	Aβ42
HC +	33.3%	0%	66.7%	0%	33.3%	0%	0%	83.3%	83.3%	16.7%	50%	0%	50%	66.7%	66.7%
HC -	33.3%	16.7%	0%	16.7%	16.7%	0%	33.3%	0%	0%	0%	0%	0%	16.7%	0%	0%
HC 0	33.3%	**83.3%**	33.3%	**83.3%**	50%	**100%**	**66.7%**	16.7%	16.7%	**83.3%**	50%	**100%**	33.3%	33.3%	33.3%
PD +	33.3%	0%	41.7%	25%	25%	0%	8.3%	41.7%	33.3%	16.7%	33.3%	0%	75%	66.7%	58.3%
PD -	25%	8.3%	0%	16.7%	8.3%	8.3%	50%	0%	0%	0%	16.7%	8.3%	8.3%	0%	0%
PD 0	41.7%	**91.7%**	**58.3%**	**58.3%**	**66.7%**	**91.7%**	41.7%	**58.3%**	**66.7%**	**83.3%**	50%	**91.7%**	16.7%	33.3%	41.7%

	**Condition:**	**Healthy Controls**										
**Serum Analyte**	**Hour:**	**0**	**1**	**2**	**4**	**6**	**10**	**12**	**16**	**20**	**24**	**26**
TNF	Mean	2.73	2.70	2.67	2.71	2.70	2.78	2.71	2.76	2.70	2.84	2.83
	SEM	0.08	0.13	0.10	0.14	0.11	0.10	0.13	0.11	0.04	0.13	0.08
IFNγ	Mean	5.49	5.28	5.03	4.64	4.96	5.28	5.47	5.68	5.39	4.72	4.62
	SEM	0.84	0.95	0.76	0.50	0.62	0.49	0.64	0.98	0.76	0.69	0.73
NGAL	Mean	1.46E+05	1.48E+05	1.47E+05	1.54E+05	1.60E+05	1.66E+05	2.26E+05	1.64E+05	1.64E+05	1.56E+05	1.59E+05
	SEM	1.08E+04	1.06E+04	8.95E+03	7.22E+03	1.07E+04	1.31E+04	6.96E+04	1.71E+04	2.18E+04	1.72E+04	2.29E+04
CRP	Mean	1.56E+06	1.53E+06	1.53E+06	1.50E+06	1.57E+06	1.56E+06	1.46E+06	1.45E+06	1.47E+06	1.52E+06	1.54E+06
	SEM	4.78E+05	4.45E+05	4.47E+05	4.11E+05	4.60E+05	4.55E+05	4.24E+05	4.38E+05	4.47E+05	4.65E+05	4.51E+05
IL-6	Mean	0.75	0.93	1.21	1.10	1.14	1.81	2.80	2.52	2.25	2.33	2.73
	SEM	0.13	0.20	0.20	0.25	0.26	0.32	0.44	1.54	0.80	0.88	1.07
IL-8	Mean	10.57	11.84	12.16	12.17	11.92	12.50	10.78	11.45	11.76	12.86	12.11
	SEM	1.48	1.87	1.96	1.83	1.67	1.55	1.44	1.81	1.62	2.39	2.18
**CSF Analyte**												
TNF	Mean	0.34	0.51	0.91	1.00	1.36	1.18	1.04	0.92	0.75	0.68	0.55
	SEM	0.10	0.08	0.18	0.19	0.29	0.17	0.37	0.25	0.17	0.12	0.07
IFNγ	Mean	0.38	0.37	0.30	0.65	0.21	1.58	1.96	1.91	3.12	2.92	2.90
	SEM	0.08	0.09	0.15	0.49	0.12	1.14	1.53	1.22	1.61	1.40	1.28
NGAL	Mean	1.95E+03	1.85E+03	1.92E+03	2.01E+03	1.89E+03	2.20E+03	1.99E+03	2.21E+03	2.38E+03	2.35E+03	2.33E+03
	SEM	65.87	79.83	111.60	186.20	178.99	160.11	193.14	225.51	283.34	307.99	303.94
CRP	Mean	3.78E+03	3.49E+03	3.96E+03	4.51E+03	4.13E+03	4.24E+03	3.93E+03	3.77E+03	3.70E+03	3.54E+03	3.51E+03
	SEM	1.29E+03	1.20E+03	1.46E+03	1.74E+03	1.59E+03	1.53E+03	1.47E+03	1.28E+03	1.21E+03	1.18E+03	1.18E+03
IL-6	Mean	1.83	2.70	4.58	8.91	11.11	16.30	14.20	17.58	19.50	15.75	13.93
	SEM	0.68	1.42	1.75	1.90	1.89	3.37	3.18	3.72	4.49	3.30	2.77
IL-8	Mean	41.61	43.69	115.18	450.78	1517.26	7985.04	2759.34	923.85	579.63	344.84	294.47
	SEM	3.69	4.94	26.41	100.15	973.42	7334.99	2224.01	508.90	240.71	99.96	92.56
αSyn	Mean	1.54E+03	1.60E+03	1.69E+03	2.05E+03	1.94E+03	1.97E+03	2.04E+03	2.15E+03	2.10E+03	2.34E+03	2.31E+03
	SEM	168.02	168.76	178.25	336.96	263.38	234.82	216.65	255.17	231.12	510.44	517.45
Aβ40	Mean	5.03E+03	5.27E+03	5.82E+03	6.11E+03	6.29E+03	6.73E+03	6.93E+03	7.49E+03	7.51E+03	7.24E+03	7.11E+03
	SEM	595.87	554.70	541.30	646.01	535.53	619.72	493.56	735.43	744.94	709.58	736.16
Aβ42	Mean	1.35E+03	1.42E+03	1.57E+03	1.62E+03	1.64E+03	1.71E+03	1.75E+03	1.91E+03	1.87E+03	1.86E+03	1.79E+03
	SEM	218.91	233.44	236.04	255.02	220.36	220.27	198.21	237.37	175.22	204.57	220.66

	**Condition:**	**Parkinson's Disease**										
**Serum Analyte**	**Hour:**	**0**	**1**	**2**	**4**	**6**	**10**	**12**	**16**	**20**	**24**	**26**
TNF	Mean	1.81	1.74	1.77	1.71	1.79	1.73	1.70	1.85	1.75	1.74	1.78
	SEM	0.18	0.15	0.15	0.14	0.15	0.13	0.13	0.16	0.13	0.16	0.14
IFNγ	Mean	5.25	4.66	4.33	3.90	3.74	3.56	3.62	3.94	3.57	3.25	3.00
	SEM	1.75	1.31	1.20	1.01	0.81	0.53	0.52	0.62	0.51	0.44	0.38
NGAL	Mean	2.09E+05	1.96E+05	2.13E+05	2.05E+05	2.10E+05	2.22E+05	2.15E+05	2.15E+05	1.93E+05	2.06E+05	2.17E+05
	SEM	1.54E+04	1.51E+04	1.55E+04	1.67E+04	1.17E+04	1.90E+04	1.52E+04	1.79E+04	2.26E+04	2.26E+04	1.53E+04
CRP	Mean	1.45E+06	1.36E+06	1.44E+06	1.37E+06	1.38E+06	1.41E+06	1.37E+06	1.40E+06	1.33E+06	1.56E+06	1.73E+06
	SEM	5.43E+05	4.75E+05	5.19E+05	4.93E+05	4.92E+05	4.65E+05	4.39E+05	4.08E+05	3.92E+05	4.25E+05	4.76E+05
IL-6	Mean	0.79	0.95	1.70	1.56	1.57	2.16	3.33	2.74	3.62	4.94	4.30
	SEM	0.09	0.12	0.42	0.28	0.35	0.55	0.74	0.58	0.73	1.67	0.97
IL-8	Mean	11.09	10.43	10.00	9.90	9.78	9.94	9.99	10.24	10.37	10.86	9.89
	SEM	2.34	2.58	2.60	2.56	2.38	2.09	2.49	2.11	2.18	2.29	2.11
**CSF Analyte**												
TNF	Mean	0.21	0.63	1.28	1.26	1.11	1.48	0.78	0.87	0.67	0.59	0.47
	SEM	0.03	0.08	0.26	0.24	0.18	0.45	0.14	0.18	0.13	0.09	0.07
IFNγ	Mean	0.21	0.28	0.17	0.11	0.17	0.37	0.40	2.47	3.16	5.21	3.45
	SEM	0.04	0.07	0.05	0.04	0.06	0.20	0.24	2.15	2.74	4.65	2.82
NGAL	Mean	2.12E+03	2.12E+03	2.01E+03	2.00E+03	2.00E+03	2.15E+03	2.05E+03	2.38E+03	2.29E+03	2.35E+03	2.37E+03
	SEM	265.89	331.17	287.94	219.75	179.89	260.08	168.31	291.09	290.43	337.50	276.51
CRP	Mean	3.76E+03	3.90E+03	3.65E+03	3.53E+03	3.32E+03	3.22E+03	3.06E+03	2.95E+03	2.93E+03	3.14E+03	3.59E+03
	SEM	1.28E+03	1.46E+03	1.37E+03	1.37E+03	1.33E+03	1.35E+03	1.29E+03	1.07E+03	1.20E+03	1.36E+03	1.74E+03
IL-6	Mean	1.93	2.24	3.55	10.09	17.74	27.27	15.86	21.00	16.31	12.24	10.14
	SEM	0.28	0.32	0.85	3.46	8.07	16.60	6.33	9.43	7.45	5.01	3.31
IL-8	Mean	38.35	43.05	74.56	298.81	516.52	529.46	376.91	316.62	246.08	159.24	159.24
	SEM	3.30	4.40	9.18	57.05	138.52	156.65	116.69	105.43	80.28	32.70	29.68
αSyn	Mean	1.14E+03	1.14E+03	1.23E+03	1.40E+03	1.53E+03	1.69E+03	1.81E+03	1.93E+03	2.00E+03	2.17E+03	2.23E+03
	SEM	118.97	92.45	113.63	130.80	153.88	180.62	216.23	243.78	260.97	296.95	304.77
Aβ40	Mean	3.75E+03	4.14E+03	4.22E+03	4.71E+03	4.98E+03	5.57E+03	5.94E+03	6.26E+03	6.51E+03	6.54E+03	6.78E+03
	SEM	316.54	292.70	355.82	359.07	448.29	524.20	575.86	716.46	806.80	847.75	853.09
Aβ42	Mean	8.70E+02	1.09E+03	1.10E+03	1.26E+03	1.40E+03	1.50E+03	1.62E+03	1.70E+03	1.76E+03	1.83E+03	1.87E+03
	SEM	77.47	125.32	122.03	152.75	179.92	199.10	220.05	246.99	299.47	320.83	308.10

The most stable analytes across time in a majority of HC and PD subjects were serum IFNγ, IL-8, & TNF and CSF IL-8, & TNF. Values denote the percentage of individuals per group (HC; n=6, PD; n=12) that display a significantly positive (+), negative (-), or no association (θ; i.e., were stable) across time. Analytes that are $\geq$ 51% stable (θ) are in bold (top). All analytes are presented as the mean pg/ml and SEM for healthy control and Parkinson's disease subjects across the day (bottom).
