# Supplementary material for: Candidate inflammatory biomarkers display unique relationships with alpha-synuclein and correlate with measures of disease severity in subjects with Parkinson’s disease
Source: J Neuroinflammation. 2017 Aug 18;14:164. doi: 10.1186/s12974-017-0935-1 (PMC5563061; doi:10.1186/s12974-017-0935-1)
Supplement: Supplementary file 2 — Serum TNF and NGAL are significantly different between PD and HC at time 0. (PDF 561 kb) [file 12974_2017_935_MOESM2_ESM.pdf]

**Supplementary Table 2. Serum TNF and NGAL are significantly different between PD and HC at Time 0.**

| | Median (pg/ml) | | Sum of Ranks | | | |
|---|---|---|---|---|---|---|
| Serum Analyte | HC | PD | HC | PD | Mann-Whitney U | p value |
| TNF | 2.72 | 1.66 | 83 | 88 | 10 | 0.01 |
| IFNγ | 5.34 | 3.60 | 70 | 101 | 23 | 0.25 |
| NGAL | 140572 | 208101 | 31 | 140 | 10 | 0.01 |
| CRP | 1245249 | 1101193 | 62 | 109 | 31 | 0.68 |
| IL-6 | 0.69 | 0.89 | 51 | 120 | 30 | 0.62 |
| IL-8 | 10.90 | 9.26 | 65 | 106 | 28 | 0.49 |
| CSF Analyte | | | | | | |
| TNF | 0.28 | 0.23 | 68 | 103 | 25 | 0.34 |
| IFNγ | 0.41 | 0.17 | 76 | 95 | 17 | 0.08 |
| NGAL | 1939 | 1933 | 79 | 221 | 50 | 0.81 |
| CRP | 3145 | 2266 | 60 | 111 | 33 | 0.82 |
| IL-6 | 1.37 | 1.76 | 45 | 126 | 24 | 0.29 |
| IL-8 | 40.27 | 36.75 | 65 | 106 | 28 | 0.49 |

Mann-Whitney U Sum of Ranks non-parametric tests determined that serum TNF and serum NGAL were the only inflammatory proteins that differed between PD and HC.
