# Supplementary material for: Candidate inflammatory biomarkers display unique relationships with alpha-synuclein and correlate with measures of disease severity in subjects with Parkinson’s disease
Source: J Neuroinflammation. 2017 Aug 18;14:164. doi: 10.1186/s12974-017-0935-1 (PMC5563061; doi:10.1186/s12974-017-0935-1)
Supplement: Supplementary file 3 — Serum TNF, IFNγ, and NGAL are different between PD and HC irrespective of time. (PDF 1693 kb) [file 12974_2017_935_MOESM3_ESM.pdf]

Supplementary Table 3. Serum TNF, IFNγ, and NGAL are significantly different between PD and HC and demonstrate minimal variability across a 24-hour period.

	Serum						CSF					
	Condition df = 1,16		Time of Day df = 10,160		Interaction df = 10,160		Condition df = 1,16		Time of Day df = 10,154		Interaction df = 10, 154	
	F stat	p value	F stat	p value	F stat	p value	F stat	p value	F stat	p value	F stat	p value
TNF	208.58	<0.0001	0.11	1.00	0.09	1.00	0.01	0.93	4.66	<0.0001[2]	0.40	0.95
IFNγ	8.49	0.009	0.38	0.95	0.16	1.00	0.00	0.98	1.09	0.37	0.14	1.00
NGAL	25.98	<0.0001	0.73	0.69	0.50	0.89	0.33	0.58	0.70	0.73	0.07	1.00
CRP	0.13	0.72	0.03	1.00	0.02	1.00	0.60	0.45	0.04	1.00	0.05	1.00
IL-6	4.23	0.06	3.12	0.001[1]	0.45	0.92	0.13	0.72	1.85	0.06	0.20	1.00
IL-8	2.18	0.16	0.04	1.00	0.06	0.99	5.08	0.04	2.23	0.01[3]	1.79	0.07
	Serum α synuclein, $A\beta_{40}$, and $A\beta_{42}$ were not collected						Condition df = 1,16		Time of Day df = 1,160		Interaction df = 1,160	
αSyn	X	X	X	X	X	X	8.35	0.01	3.30	0.0007[4]	0.24	0.99
$A\beta_{40}$	X	X	X	X	X	X	14.69	0.001	4.11	<0.0001[4]	0.13	1.00
$A\beta_{42}$	X	X	X	X	X	X	4.61	0.05	2.15	0.02[5]	0.26	0.99

Two-way repeated measures analysis of variance by time and condition (HC and PD) for each serum and CSF analyte revealed that serum TNF, IFNγ, and NGALdiffer between HC and PD across the day. Superscripts indicate the hour of the day where significant differences occur; 1 = 0 through 10th sampling hour, 2 = 6th and 10th sampling hour, 3 = 10th sampling hour, 4 = 0 through 6th sampling hour, and 5 = 0 through 4th sampling hour. X indicates that samples were not collected or analyzed.
