# Supplementary material for: Candidate inflammatory biomarkers display unique relationships with alpha-synuclein and correlate with measures of disease severity in subjects with Parkinson’s disease
Source: J Neuroinflammation. 2017 Aug 18;14:164. doi: 10.1186/s12974-017-0935-1 (PMC5563061; doi:10.1186/s12974-017-0935-1)
Supplement: Supplementary file 4 — PD and HC subjects have different relationships between serum and CSF inflammation. (PDF 801 kb) [file 12974_2017_935_MOESM4_ESM.pdf]

Supplementary Table 4. Repeated measures ANCOVA adjusted for time revealed several unique relationships between serum and CSF inflammation that are different between HC and PD subjects.

	Condition df = 1,16		Covariate (CSF Analyte) df = 1,172		Interaction df = 1,172	
Serum Analyte	F stat	p value	F stat	p value	F stat	p value
TNF	76.68	<0.0001	0.60	0.4	0.00	1.0
IFNγ	6.85	0.02	0.09	0.8	0.11	0.7
NGAL	0.64	0.4	8.65	0.004	0.54	0.5
CRP	2.34	0.15	1318.62	< 0.0001	6.94	0.009
IL-6	13.11	0.002	11.72	0.0007	10.79	0.001
IL-8	5.27	0.04	0.21	0.6	0.46	0.5

Serum CRP and CSF CRP significantly covary in one group but not the other. Serum IL-6 and CSF IL-6 have different relationships between the two groups. Serum NGAL and CSF NGAL have a significant relationship that is not different between the two groups. Serum IFNγ, serum IL-8, and serum TNF do not covary with CSF analytes, but are different between PD and HC. Significant values are in dark font.
