# Supplementary material for: Candidate inflammatory biomarkers display unique relationships with alpha-synuclein and correlate with measures of disease severity in subjects with Parkinson’s disease
Source: J Neuroinflammation. 2017 Aug 18;14:164. doi: 10.1186/s12974-017-0935-1 (PMC5563061; doi:10.1186/s12974-017-0935-1)
Supplement: Supplementary file 5 — Summary of relationships between inflammation and Aβs and α-synuclein in PD and HC. (PDF 2346 kb) [file 12974_2017_935_MOESM5_ESM.pdf]

Supplementary Table 5. Repeated measures ANOVA adjusted for time revealed several unique relationships between serum and CSF inflammation and established biomarkers of neurodegenerative disease between PD and HC groups.

	α Syn						$A\beta_{40}$						$A\beta_{42}$					
	Condition df = 1,16		Covariate (αSyn) df = 1,172		Interaction df = 1,172		Condition df = 1,16		Covariate ($A\beta_{40}$) df = 1,172		Interaction df = 1,172		Condition df= 1,16		Covariate ($A\beta_{42}$) df = 1,172		Interaction df = 1,172	
Serum Analyte	F stat	p value	F stat	p value	F stat	p value	F stat	p value	F stat	p value	F stat	p value	F stat	p value	F stat	p value	F stat	p value
TNF	44.66	<0.0001	2.84	0.09	1.02	0.3	33.69	<0.0001	1.25	0.3	2.65	0.1	23.65	0.0002	2.80	0.1	0.22	0.6
IFNγ	1.09	0.3	14.34	0.0002	7.04	0.009	0.73	0.4	9.37	0.003	4.29	0.04	0.84	0.4	0.31	0.6	4.31	0.04
NGAL	26.72	<0.0001	0.40	0.5	11.58	0.0008	19.34	0.0004	0.01	0.9	9.67	0.002	2.20	0.2	2.56	0.1	0.08	0.8
CRP	10.05	0.006	5.34	0.02	12.8	0.0004	7.02	0.02	14.86	0.0002	7.55	0.007	21.93	0.0002	14.34	0.0002	26.66	<0.0001
IL-6	1.36	0.3	2.35	0.1	3.54	0.06	0.81	0.4	1.71	0.2	1.92	0.2	0.18	0.7	1.90	0.2	1.05	0.3
IL-8	0.17	0.7	5.67	0.02	1.02	0.3	0.80	0.4	0.41	0.5	3.87	0.05	0.09	0.8	0.20	0.7	2.28	0.1
CSF Analyte	F stat	p value	F stat	p value	F stat	p value	F stat	p value	F stat	p value	F stat	p value	F stat	p value	F stat	p value	F stat	p value
TNF	9.65	0.007	1.06	0.3	12.60	0.0005	12.72	0.003	0.02	0.9	15.39	0.0001	14.89	0.001	6.96	0.009	16.98	<0.0001
IFNγ	0.06	1.0	15.58	0.0001	0.11	0.7	1.13	0.3	12.25	0.0006	0.80	0.4	0.46	0.5	43.16	<0.0001	1.19	0.3
NGAL	0.99	0.3	0.24	0.6	0.71	0.4	4.61	0.05	3.48	0.06	4.07	0.05	4.49	0.05	6.47	0.01	3.92	0.05
CRP	7.53	0.01	7.31	0.008	10.03	0.002	5.93	0.03	18.67	<0.0001	6.50	0.01	22.68	0.0002	16.75	<0.0001	28.26	<0.0001
IL-6	0.74	0.4	2.06	0.2	1.38	0.2	0.01	0.9	2.90	0.09	0.11	0.7	5.50	0.03	10.44	0.002	8.33	0.004
IL-8	0.02	0.9	0.12	0.7	0.51	0.5	1.74	0.2	2.67	0.1	3.83	0.05	9.41	0.007	14.40	0.0002	15.97	<0.0001

With respect to CSF α synuclein, HC and PD subjects have different relationships between this biomarker and serum IFNγ, serum NGAL, serum CRP, CSF TNF, and CSF CRP. SerumIL-8 and CSF IFNγ significantly covary with α synuclein, but the relationships do not differ between groups (PD and HC). With respect to CSF Aβ40, HC and PD subjects have different relationships between this biomarker and serum IFNγ,serum NGAL, serum CRP, serum IL-8, CSF TNF, CSF NGAL, CSF CRP, and CSF IL-8. CSF IFNγ covaries with Aβ40, but the relationships do not differ between PD and HC subjects. With respect to Aβ42, HC and PD subjects have different relationships between this biomarker and serum IFNγ, serum CRP, CSF TNF, CSF NGAL, CSF CRP, CSF IL-6, and CSF IL-8. CSF IFNγ covaries with Aβ42, but the relationship does not differ between PD and HC subjects.
