# Supplementary figures and images for: Candidate inflammatory biomarkers display unique relationships with alpha-synuclein and correlate with measures of disease severity in subjects with Parkinson’s disease
Source: J Neuroinflammation. 2017 Aug 18;14:164. doi: 10.1186/s12974-017-0935-1 (PMC5563061; doi:10.1186/s12974-017-0935-1)

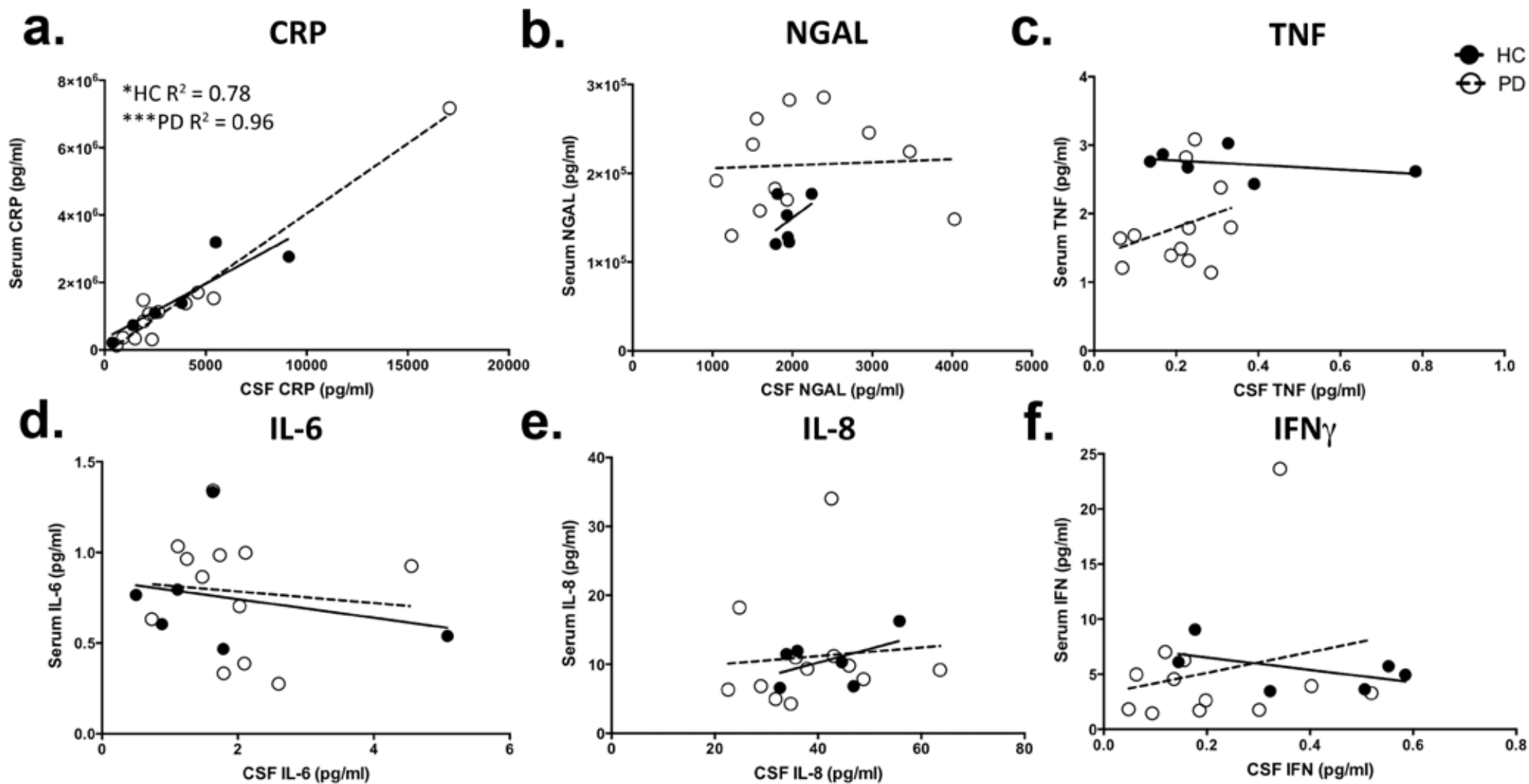

Supplementary Figure 1

Supplement: Supplementary file 6 — Serum and CSF CRP levels are positively correlated in HC and PD at baseline. The relationship between serum and CSF inflammatory factors was analyzed using samples from the first collection period (baseline; time 0). There was no significant correlation between serum and CSF levels of any factor except CRP (a). Serum and CSF CRP significantly correlate in HC (slope = 325 ± 86.9, F (1, 4) = 14.0; p = 0.02) and in PD (slope = 415.5 ± 26.6, F (1, 10) = 243.8; p < 0.0001), but the slopes do not differ between HC and PD (t(16) = 1.28; p = 0.22). Serum and CSF NGAL (HC; slope = 66.57 ± 64.63, R 2 = 0.17, F (1,4) = 0.80; p = 0.42 and PD; slope = 3.44 ± 18.28, R 2 = 0.003, F (1,10) = 0.04; p = 0.85) (b), TNF (HC; slope = −0.33 ± 0.40, R 2 = 0.15, F (1,4) = 0.68; p = 0.46 and PD; slope = 2.15 ± 2.13, R 2 = 0.09, F (1,10) = 1.02; p = 0.34) (c), IL-6 (HC; slope = −0.05 ± 0.09, R 2 = 0.07, F (1,4) = 0.32; p = 0.60 and PD; slope = −0.03 ± 0.11, R2 = 0.009, F (1,10) = 0.09; p = 0.77) (d), IL-8 (HC; slope = 0.20 ± 0.17, R 2 = 0.25, F (1,4) = 1.31; p = 0.32 and PD; slope = 0.06 ± 0.22, R 2 = 0.01, F (1,10) = 0.08; p = 0.79) (e), and IFNγ (HC; slope = −5.64 ± 4.78, R 2 = 0.28, F (1,4) = 1.59; p = 0.28 and PD; slope = 9.42 ± 12.77, R 2 = 0.05, F (1,10) = 0.54; p = 0.48) (f) do not correlate at baseline. (PDF 74 kb) [file 12974_2017_935_MOESM6_ESM.pdf]

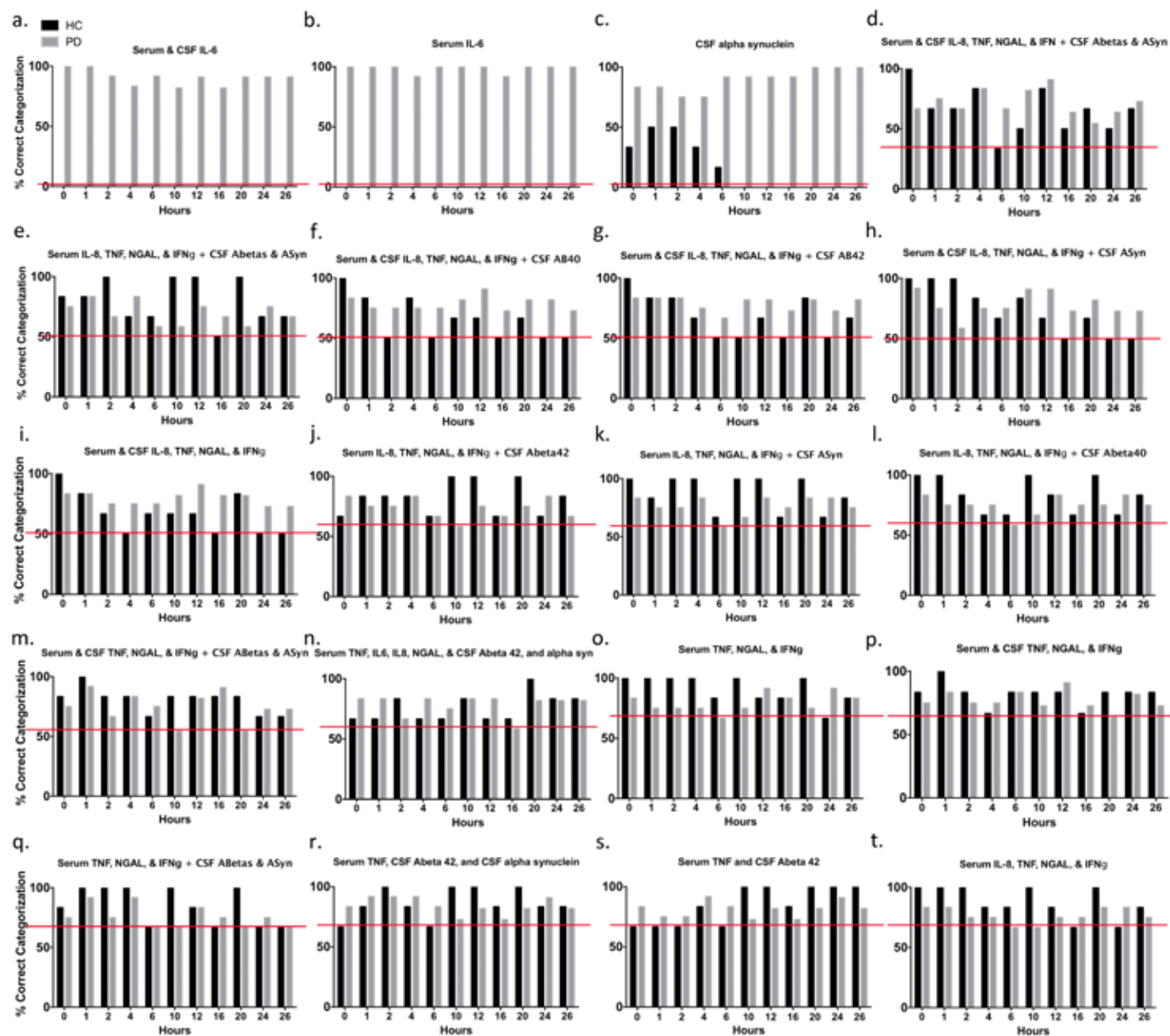

Supplementary Figure 2

Supplement: Supplementary file 9 — Linear discriminant analysis reveals sensitivity and selectivity of analytes in discriminating between HC and PD. Serum and CSF levels of IL-6 (a), serum IL-6 (b). Serum and CSF IL-8, TNF, NGAL, and IFNγ, and CSF α-synuclein, Aβ40 and Aβ42 (d), serum IL-8, TNF, NGAL, IFNγ, and CSF α-synuclein, Aβ40, and Aβ42 (e), serum and CSF IL-8, TNF, NGAL, IFNγ, and CSF Aβ40 (f), serum and CSF IL-8, TNF, NGAL, IFNγ, and CSF Aβ42 (g), serum and CSF IL-8, TNF, NGAL, IFNγ, and CSF α-synuclein (h), serum and CSF IL-8, TNF, NGAL, IFNγ (i), serum IL-8, TNF, NGAL, IFNγ, and CSF Aβ42 (j), serum IL-8, TNF, NGAL, IFNγ, and CSF α-synuclein (k), serum IL-8, TNF, NGAL, IFNγ, and CSF Aβ40 (l), serum and CSF TNF, NGAL, IFNγ, and CSF α-synuclein, Aβ40, and Aβ42 (m), serum TNF, NGAL, IFNγ, and CSF α-synuclein, and Aβ42 (n), serum TNF, NGAL, and IFNγ (o), serum and CSF TNF, NGAL, and IFNγ (p), serum TNF, NGAL, and IFNγ and CSF α-synuclein, Aβ40, and Aβ42 (q), serum TNF and CSF α-synuclein, and Aβ42 (r), serum TNF and CSF Aβ42 (s), serum IL-8, TNF, NGAL, and IFNγ (t). The x axis is the percentage of correct categorization into a group, and the red line is set at the lowest percentage of correct categorizations for the factor or factors considered. The y axis is the collection hour. (PDF 239 kb) [file 12974_2017_935_MOESM9_ESM.pdf]
