# Supplementary material for: Candidate inflammatory biomarkers display unique relationships with alpha-synuclein and correlate with measures of disease severity in subjects with Parkinson’s disease
Source: J Neuroinflammation. 2017 Aug 18;14:164. doi: 10.1186/s12974-017-0935-1 (PMC5563061; doi:10.1186/s12974-017-0935-1)
Supplement: Supplementary file 7 — Baseline CSF α-synuclein and Aβs and CSF and serum inflammatory proteins between PD and HCs. (PDF 1977 kb) [file 12974_2017_935_MOESM7_ESM.pdf]

Supplementary Table 6. The relationship between the baseline (Time 0) expression of CNS proteins associated with neurodegenerative disease (α-synuclein, $A\beta_{40}$, and $A\beta_{42}$) and peripheral (serum) and central (CSF) inflammatory proteins between PD and HC groups.

	α Syn					
	Slope		R^2		ANOVA stats	
Serum Analyte	HC	PD	HC	PD	HC	PD
TNF	-0.000 ± 0.000	-0.000 ± 0.000	0.069	0.147	F (1,4) = 0.290; p = 0.62	F(1,10) = 1.72; p = 0.22
IFNγ	-0.001 ± 0.002	-0.007 ± 0.004	0.036	0.233	F (1,4) = 0.150; p = 0.72	F(1,10) = 3.045; p = 0.11
NGAL	10.93 ± 31.56	-2.607 ± 40.91	0.029	0.000	F (1,4) = 0.120; p = 0.75	F (1,10) = 0.004; p = 0.95
CRP	1938 ± 1040	-242.7 ± 1441	0.465	0.003	F (1,4) = 3.471; p = 0.14	F (1,10) = 0.028; p = 0.87
IL-6	-0.000± 0.000	-0.000 ± 0.000	0.166	0.022	F (1,4) = 0.794; p = 0.42	F (1,10) = 0.220; p = 0.65
IL-8	-0.001 ± 0.004	-0.008 ± 0.006	0.005	0.164	F (1,4) = 0.019; p = 0.90	F (1,10) = 1.961; p = 0.19
CSF Analyte	HC	PD	HC	PD	HC	PD
TNF	0.000 ± 0.000	-0.000± 0.000	0.047	0.006	F (1,4) = 0.197; p = 0.68	F (1,10) = 0.058; p = 0.82
IFNγ	**0.000± 0.983**	-0.000 ± 0.000	**0.825**	0.088	**F (1,4) = 18.88; p = 0.01**	F (1,10) = 0.966; p = 0.35
NGAL	0.012 ± 0.196	**1.642 ± 0.480**	0.001	**0.540**	F (1,4) = 0.004; p = 0.95	**F (1,10) = 11.73; p =0.007**
CRP	5.085 ± 2.896	0.767 ± 3.396	0.435	0.005	F (1,4) = 3.083; p = 0.15	F (1,10) = 0.051; p 0.83
IL-6	0.001 ± 0.002	0.000 ± 0.001	0.018	0.001	F (1,4) = 0.075; p = 0.80	F (1,10) = 0.097; p =0.76
IL-8	0.008 ± 0.010	0.005 ± 0.009	0.160	0.030	F(1,4)=0.730; p =0.40	F(1,10)=0.310; p =0.60

	Aβ40					
	Slope		R^2		ANOVA stats	
Serum Analyte	HC	PD	HC	PD	HC	PD
TNF	-0.000 ± 0.000	-0.000± 0.000	0.013	0.029	F (1,4) = 0.053; p = 0.83	F (1,10) = 0.301; p = 0.60
IFNγ	-0.000 ± 0.001	-0.003 ± 0.001	0.006	0.273	F (1,4) = 0.024; p = 0.88	F (1,10) = 3.760; p = 0.08
NGAL	-0.766 ± 9.024	2.707 ± 15.35	0.002	0.003	F (1,4) = 0.007; p = 0.94	F (1,10) = 0.031; p = 0.86
CRP	**664.6 ± 224.1**	632.5 ± 504.2	**0.688**	0.136	**F (1,4) = 8.799; p = 0.04**	F (1,10) = 1.574; p = 0.24
IL-6	-0.000 ± 0.000	-0.000 ± 0.000	0.052	0.000	F (1,4) = 0.220; p = 0.66	F (1,10) = 0.001; p = 0.97
IL-8	-0.001 ± 0.001	-0.001 ± 0.001	0.115	0.039	F (1,4) = 0.520; p = 0.51	F (1,10) = 0.360; p = 0.56
CSF Analyte	HC	PD	HC	PD	HC	PD
TNF	-0.000± 0.000	0.000± 0.000	0.004	0.046	F (1,4) = 0.015; p = 0.91	F (1,10) = 0.486; p = 0.50
IFNγ	0.00 ± 0.000	-0.000± 0.000	0.617	0.023	F (1,4) = 6.444; p = 0.06	F (1,10) = 0.235; p = 0.64
NGAL	-0.029 ± 0.053	**0.511 ± 0.211**	0.068	**0.369**	F (1,4) = 0.292; p = 0.62	**F (1,10) = 5.858; p = 0.04**
CRP	**1.775 ± 0.627**	2.049 ± 1.103	**0.668**	0.256	**F (1,4) = 8.029; p = 0.05**	F (1,10) = 3.448; p = 0.09
IL-6	-0.000 ± 0.001	0.000 ± 0.000	0.023	0.100	F (1,4) = 0.094; p =0.77	F (1,10) = 1.11; p = 0.32
IL-8	0.000 ± 0.003	0.004 ± 0.003	0.033	0.185	F(1,4) = 0.140; p = 0.73	F(1,10) = 2.270; p = 0.16

	Aβ42					
	Slope		R^2		ANOVA stats	
Serum Analyte	HC	PD	HC	PD	HC	PD
TNF	0.000 ± 0.000	-0.000 ± 0.001	0.005	0.165	F (1,4) = 0.020; p = 0.90	F (1,10) =1.978; p = 0.19
IFNγ	0.000 ± 0.002	-0.007 ± 0.007	0.025	0.109	F (1,4) = 0.102; p = 0.78	F (1,10) = 1.217; p = 0.30
NGAL	-15.35 ± 23.36	42.73 ± 61.36	0.097	0.046	F (1,4) = 0.432; p = 0.55	F (1,10) = 0.485; p = 0.50
CRP	1692 ± 688.8	542.3 ± 2210	0.601	0.006	F (1,4) = 6.035; p = 0.07	F (1,10) = 0.060; p = 0.81
IL-6	-0.000 ± 0.000	-0.000 ± 0.000	0.006	0.001	F (1,4) = 0.025; p = 0.88	F (1,10) = 0.013; p =0.91
IL-8	-0.004 ± 0.003	-0.013 ± 0.009	0.349	0.191	F (1,4) = 2.142; p = 0.22	F (1,10) = 2.355; p = 0.16
CSF Analyte	HC	PD	HC	PD	HC	PD
TNF	-0.000 ± 0.000	0.000 ± 0.000	0.096	0.245	F (1,4) = 0.426; p = 0.55	F (1,10) = 3.241; p = 0.10
IFNγ	0.000± 0.000	-0.000± 0.000	0.254	0.000	F (1,4) = 1.306; p = 0.31	F (1,10) = 0.001; p = 0.98
NGAL	-0.154 ± 0.129	0.977 ± 1.04	0.262	0.081	F (1,4) = 1.419; p = 0.30	F (1,10) = 0.881; p = 0.37
CRP	4.579 ± 1.872	2.659 ± 5.16	0.599	0.026	F (1,4) = 5.980; p = 0.07	F (1,10) = 0.266; p = 0.62
IL-6	-0.001 ± 0.001	-0.001 ± 0.001	0.135	0.101	F (1,4) = 0.624; p = 0.47	F (1,10) = 1.125; p = 0.32
IL-8	-0.000 ± 0.008	0.007 ± 0.013	0.003	0.020	F(1,4) = 0.01; p = 0.92	F(1,10) = 0.240; p = 0.63

PD subjects demonstrate a relationship between CSF NGAL and CSF α-synuclein as well as CSF NGAL and CSF $A\beta_{40}$, while HC subjects do not. HC subjects demonstrate a relationship between CSF IFNγ and CSF α-synuclein, CSF CRP and $A\beta_{40}$, and serum CRP and $A\beta_{40}$, while PD subjects do not. There were no other significant relationships at Time 0 (see Figure 3).
