# Supplementary material for: Candidate inflammatory biomarkers display unique relationships with alpha-synuclein and correlate with measures of disease severity in subjects with Parkinson’s disease
Source: J Neuroinflammation. 2017 Aug 18;14:164. doi: 10.1186/s12974-017-0935-1 (PMC5563061; doi:10.1186/s12974-017-0935-1)
Supplement: Supplementary file 8 — CSF IFNγ and serum IL-8 positively correlated with UPDRS components at baseline in PD subjects. (PDF 563 kb) [file 12974_2017_935_MOESM8_ESM.pdf]

Supplementary Table 7. CSF IFNγ positively correlated with the Unified Parkinson's Disease Rating Score (UPDRS) components, and serum IL-8 positively correlated with Activities of Daily Living Score (ADLS) at Time 0 in PD subjects.

	UPDRS Total Score		Mentation, Behavior, & Mood		Activities of Daily Living		Motor Examination		Complication of Therapy		Years since diagnosis	
	R^2	p value	R^2	p value	R^2	p value	R^2	p value	R^2	p value	R^2	p value
Serum Analyte												
IL-6	0.15	0.21	0.27	0.08	0.05	0.49	0.08	0.39	0.28	0.08	0.03	0.58
IL-8	0.26	0.09	0.02	0.69	0.35	0.04	0.11	0.30	0.26	0.09	0.03	0.60
IFNγ	0.005	0.82	0.01	0.76	0.002	0.90	0.03	0.56	0.003	0.87	0.002	0.89
TNF	0.08	0.37	0.05	0.49	0.04	0.52	0.12	0.26	0.04	0.52	0.008	0.78
NGAL	0.02	0.69	0.0002	0.96	0.04	0.52	0.01	0.74	0.08	0.37	0.07	0.41
CRP	0.008	0.79	0.0004	0.95	0.07	0.42	0.0008	0.93	0.008	0.78	0.02	0.67
CSF Analyte												
IL-6	0.02	0.66	0.08	0.39	0.0002	0.96	0.004	0.85	0.09	0.35	0.16	0.20
IL-8	0.05	0.49	0.06	0.45	0.09	0.35	0.04	0.51	0.0003	0.96	0.003	0.86
IFNγ	0.57	0.004	0.22	0.12	0.60	0.003	0.27	0.09	0.57	0.005	0.03	0.61
TNF	0.14	0.24	0.16	0.20	0.15	0.22	0.008	0.78	0.23	0.11	0.00003	0.99
NGAL	0.02	0.64	0.004	0.85	0.003	0.86	0.02	0.69	0.17	0.18	0.007	0.80
CRP	0.007	0.79	0.0004	0.95	0.05	0.51	0.00006	0.98	0.0002	0.97	0.01	0.68
