# Supplementary material for: Candidate inflammatory biomarkers display unique relationships with alpha-synuclein and correlate with measures of disease severity in subjects with Parkinson’s disease
Source: J Neuroinflammation. 2017 Aug 18;14:164. doi: 10.1186/s12974-017-0935-1 (PMC5563061; doi:10.1186/s12974-017-0935-1)
Supplement: Supplementary file 10 — Subject demographics. (PDF 411 kb) [file 12974_2017_935_MOESM10_ESM.pdf]

Supplementary Table 8. Subject Demographics

	Age (years)		Weight (kg)		BMI (kg/m^2)		Sex	Ethnicity		Race		
	Mean	SD	Mean	SD	Mean	SD	Male	Hispanic or Latino	Not Hispanic or Latino	Black or African American	Asian	White
**PD**	51.8	7.96	82.43	16.05	26.53	3.85	75%	0%	100%	0%	0%	100%
**HC**	53.3	11.41	81.07	15.48	27.28	2.94	66.7%	33.3%	66.7%	16.67%	33.3%	50%
