# Supplementary material for: Candidate inflammatory biomarkers display unique relationships with alpha-synuclein and correlate with measures of disease severity in subjects with Parkinson’s disease
Source: J Neuroinflammation. 2017 Aug 18;14:164. doi: 10.1186/s12974-017-0935-1 (PMC5563061; doi:10.1186/s12974-017-0935-1)
Supplement: Supplementary file 11 — Inclusion and exclusion criteria summary. (PDF 1759 kb) [file 12974_2017_935_MOESM11_ESM.pdf]

Supplementary Table 9. Inclusion and Exclusion criteria summary

PD	HC	Inclusion criterion	PD	HC	Exclusion criterion
●	●	≥ 40 years of age	●	●	Current treatment with anti-coagulants
●	●	Body Mass Index (BMI) between 18-32 km/m^2	●	●	Use of investigational drugs or devices within 30 days or 5 half-lives (whichever is longer) before sample collection (dietary supplements taken outside of a clinical trial are not exclusionary; e.g., coenzyme Q10)
●	●	Weight of > 50 kg (110 lbs)	●	●	Any medical assessment, condition, or circumstance making patient unsuitable for the study
●	●	Signed Informed Consent	●	●	lower spinal malformations, local infection, or other spinal abnormality that would exclude lumbar puncture
●		≥ 30 years of age at time of PD diagnosis	●	●	History of febrile illness within 5 days of sample collection
●		Resting tremor, bradykinesia, rigidity (must have either resting tremor or bradykinesia)	●	●	History of regular alcohol consumption exceeding 14 units/ week (1 unit = equivalent of 83 ml 12 % strength wine)
●		PD for ≤ 10 years at screening	●	●	Urine test positive for drugs of abuse or alcohol at screening and prior to sample collection
●		Hoehn Yahr stage I - III	●	●	Pregnancy test positive
●		Taking PD medication including amantadine, DA agonists, L-DOPA, and/ or MAO-B inhibitors (must have been on a stable dose for 4 weeks prior to sample collection)	●	●	Use of prescription or non-prescription drugs, vitamins, or dietary supplement within 7 days or 5 half lives prior to sample collection deemed by the PI to interfere with the study
			●	●	Herbal supplements and hormonal contraception or replacement of any kind not discontinued 28 days prior to sample collection as deemed appropriate by the PI
			●	●	Donated > 500 ml of blood within 5 days prior to sample collection
			●	●	Positive for Hepatitis B, Hepatitis C, or HIV at screening
			●	●	Strenuous exercise (> 5 hours / week running, swimming, or similar physical activity) within 72 hours prior to sample collection
			●	●	Allergy to Lidocaine (xylocaine) or its derivatives
			●	●	Evidence or history of significant active bleeding or coagulation disorder
			●	●	Evidence or history of back pain or injury deemed clinically significant by PI
			●	●	Evidence or history of migraines deemed clinically significant by PI
				●	Current or active clinically significant neurological disorder
				●	First degree relative with idiopathic PD (parent, sibling, or child)
				●	Currently taking DA agonists or other PD medications

Dots indicate use of criterion for subject inclusion or exclusion.
